# Precursor-dependent structural diversity in luminescent carbonized polymer dots (CPDs): the nomenclature

**DOI:** 10.1038/s41377-021-00579-6

**Published:** 2021-07-12

**Authors:** Qingsen Zeng, Tanglue Feng, Songyuan Tao, Shoujun Zhu, Bai Yang

**Affiliations:** State Key Laboratory of Supramolecular Structure and Materials, College of Chemistry, Jilin University, Changchun, 130012 China

**Keywords:** Nanoparticles, Polymers

## Abstract

Carbon dots (CDs) have received immense attention in the last decade because they are easy-to-prepare, nontoxic, and tailorable carbon-based fluorescent nanomaterials. CDs can be categorized into three subgroups based on their morphology and chemical structure: graphene quantum dots (GQDs), carbon quantum dots (CQDs), and carbonized polymer dots (CPDs). The detailed structures of the materials can vary significantly, even within the same category. This property is particularly predominant in chemically synthesized CPDs, as their formation proceeds via the polymerization–carbonization of molecules or polymer precursors. Abundant precursors endow CPDs with versatile structures and properties. A wide variety of carbon nanomaterials can be grouped under the category of CPDs because of their observed diversity. It is important to understand the precursor-dependent structural diversity observed in CPDs. Appropriate nomenclature for all classes and types of CPDs is proposed for the better utilization of these emerging materials.

## Abstract (web summary)

A wide variety of carbon nanomaterials can be grouped under the category of carbonized polymer dots (CPDs) because of their observed structural diversity. Nomenclature is thus proposed for the better identification of various CPDs materials.

## Graphical abstract

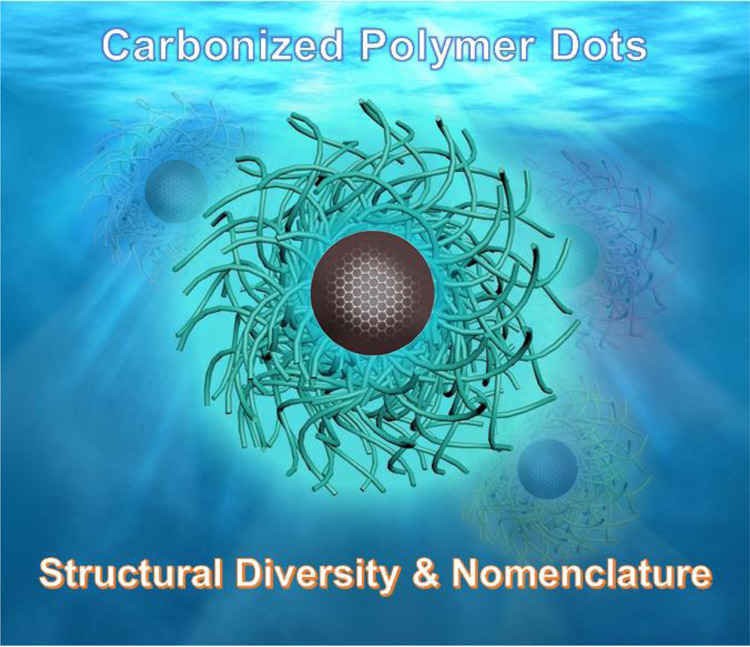

## Introduction

Carbon dots (CDs) are a class of emerging fluorescent materials that exhibit high photostability, excellent photoelectric/optical properties and low toxicity^1–6^. They have been widely used to fabricate biomedical appliances^7,8^, optoelectronic devices^9,10^, and sensors^11^ and have also been used in the fields of energy conversion^12^, anti-counterfeiting technology^13–15^, and catalysis^16–18^. CDs are spheroidal carbon nanoparticles horizontally smaller than 10 nm and even shorter vertically^12^. Various synthetic routes have been followed for the synthesis of CDs; these routes can produce CDs with diverse chemical structures, physical/chemical properties, and functions. The two widely used techniques for fabricating CDs are top-down cutting and bottom-up synthesis methods^19^. In the top-down synthesis method, bulk carbon resources exhibiting a perfect sp^2^ carbon structure are split into small fragments by the processes of oxidation and exfoliation. The final CDs have a graphite-type carbon skeleton and can be identified as graphene quantum dots (GQDs) or carbon quantum dots (CQDs)^20^. GQDs are anisotropic in nature and contain very few layers of sp^2^-graphene fragments with sufficient numbers of surface/edge groups. They have tunable photoluminescence (PL) emission dominated by quantum confinement and edge effects^20^. CQDs are typically highly crystalline spherical sp^2^-carbon structures and exhibit the property of size-dependent PL emission^20^.

In recent years, researchers have attempted the chemical synthesis of CDs starting from molecules or polymer precursors (bottom-up routes) to gain better control of the final materials^21^. The precursors generally bear polymerizable moieties such as hydroxyl groups (−OH), carboxyl groups (−COOH), amino groups (−NH_2_), and double bonds (−C=C) in their skeleton that can participate in condensation/addition–polymerization reactions. Precursors undergo polymerization/crosslinking and carbonization to form a carbonized polymeric structure. Except for a few CQDs that are produced from highly symmetrical conjugated molecules such as polyphenol and aromatic amine^22^, the majority of the CDs prepared through the bottom-up synthesis method from asymmetrical precursors belong to the category of carbonized polymer dots (CPDs)^20^. The structure of CPDs consists of an inner carbogenic core (sp^2^-hybridized carbon structures within sp^3^-hybridized frameworks) and abundant surface nitrogen/oxygen-based groups alongside even short polymer chain-like structures, which is attributed to the unique synthetic route of the polymerization–carbonization process^12^. Compared with the top-down fabrication of CQDs and GQDs, bottom-up synthesis can be more systematic and logical in controlling the optical properties of CPDs during the polymerization–carbonization process; therefore, CPDs generally exhibit outstanding optical properties (e.g., high photoluminescence quantum yields (PL QY) and easy-to-tune emission). The precursors used to dictate the atomic arrangement in the CPDs. Thus, the chemical structures of CPDs can be easily tailored by selecting the appropriate precursors. The precursors can be nonaromatic compounds, aromatic compounds, metal-containing molecules, polymers, or biomass materials. The structural and functional diversities in CPD materials can be achieved using various types of precursors. However, there is no nomenclature to index such a diverse material system. Therefore, a method of naming the CPDs should be proposed to better index and understand this new class of materials.

In this *Perspective*, we outlined the steps of the polymerization–carbonization process followed for the development of CPDs. The polymerization–carbonization route is responsible for the precursor-dependent diversity and the property of intrinsic polydispersity observed in CPDs. The precursor-dependent structural, optical and functional diversity are highlighted. Based on the structural features of CPDs, this material system can be indexed according to the precursors. Each CPD material has been named after its precursor. Seven rules have been laid out to determine the priority order of the precursors. Important information on the local micro-nanostructures, properties, and fields of application of CPDs can be communicated through their names. This system of nomenclature can be used to conveniently identify different CPD materials. The establishment of a nomenclature system for CPDs can potentially be used to index the library of CPD materials.

## Structural and optical properties of CPDs throughout the polymerization–carbonization process

**Fig. 1:**
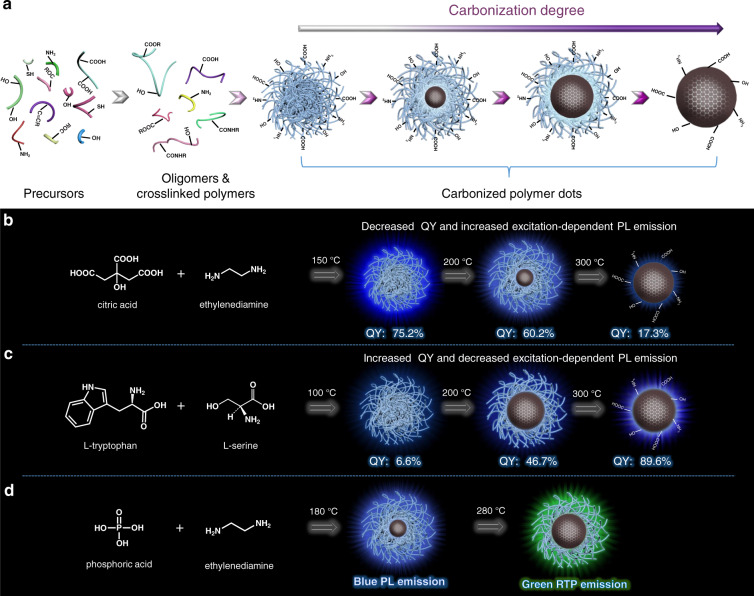
Polymerization–carbonization process for the synthesis of CPDs. **a** Schematic representation of the hydrothermal–polymerization–carbonization process for the preparation of CPDs. Influence of the carbonization process on the optical emission properties of typical CPD systems prepared from (**b**) CA and EDA, (**c**) L-tryptophan and L-serine, and (**d**) PA and EDA.

The study of chemically synthesized CDs (bottom-up pathway) began in 2008^23^. Over the years, the concept of CPDs has undergone subtle changes with a deepening understanding of its formation process. The modified concept of CPDs (originally known as polymer CDs^24^) has been introduced by our group. The term CPDs reveals the formation mechanism of CDs that were synthesized through the bottom-up pathway via polymerization and carbonization. The structural diversity of CPDs actually originates from the polymerization–carbonization reaction pathway, which is similar to the polymerization reaction process. The complexity of the polymerization–carbonization reaction pathway can be attributed to the simultaneous/subsequent carbonization reaction. We have considered the widely used hydrothermal method (a hydrothermal–polymerization–carbonization (HTPC) reaction) to describe this process (Fig. 1a)^24^. Recently, the addition–polymerization reaction (and not the common polycondensation reaction) has been developed for synthesizing the target material. This development indicates the versatility of the polymerization–carbonization method that can be used for the fabrication of different types of CPDs^25,26^. The growth of the precursors can be attributed to the progress of the intermolecular polymerization reactions, resulting in the generation of oligomers and less densely crosslinked polymer chains. These serve as building blocks for the formation of CPDs. As the polymerization reaction proceeds, rigid entanglement among the polymer chain segments can be observed, resulting in the formation of highly crosslinked polymer nanoclusters. The formation of CPDs can be observed at this stage. A simultaneous carbonization process is also observed. As the reaction temperature and reaction time increase, the number of polymeric structures gradually decreases. This trend is accompanied by the appearance of microcrystalline regions or lattices in the CPDs. The progress of the carbonization process is accompanied by the growth of the CPD core. This growth can be attributed to the consumption of surface polymer chains or precursors. The carbonization process is a combination of dehydration, decarboxylation, deamination, and/or dehydrogenation reactions. These reactions primarily depend on the molecular structures of the precursors and the reaction temperature. Therefore, an increase in the degree of carbonization results in an increase in the C content. Increased carbonization also results in the enlargement of the sp^2^ domains in the carbon core^11,27^. Completely carbonized CPDs are primarily composed of surface groups and carbon cores. Subsequent purification procedures are vital for extracting the final CPD products, including filtration, dialysis, or chromatography. These steps are necessary for removing low molecular weight fluorescent molecules or aggregated carbonaceous materials^28^. Characterization is pivotal to obtain the morphology and optical properties of CPDs^28–31^. In addition to conventional morphology and optical characterizations, new characterization techniques need to be developed to determine the chemical structure and formation mechanism of CPDs. Very recently, Prato’s Group tracked the structural phenomena during the synthesis of arginine (Arg)- and ethanediamine (EDA)-derived CPDs using a combined spectroscopic approach^32^. They clearly revealed the polymerization–carbonization process in the Arg-EDA CPD system, including four steps: (i) polymerization of small organic molecules, (ii) formation of a dense core with an extended shell, (iii) collapse of the shell, and (iv) carbonization/aromatization of the core. In addition, they demonstrated the core-shell structure and determined the respective thickness of shell and core layers through a small-angle X-ray scattering pattern. Rationally designing the core-shell structure of CPDs will be a step forward towards materials with ideal properties and functions in future research^32^.

The term CPDs reveals the formation process of polymerization and carbonization in bottom-up-synthesized CDs. This term encompasses a series of CPDs exhibiting varying degrees of carbonization (Fig. 1a). Slightly carbonized CPDs consisting of polymer chains/carbon hybrid structures and highly carbonized CPDs composed of carbon core and surface groups also fall under this category.

The degree of carbonization can be tuned and generally depends on the reaction temperature in a specific CPD system. A classical CPD model was prepared from citric acid (CA) and EDA. The CA-EDA CPDs exhibited an ultrahigh PL QY. The reaction mechanism for the formation of these CPDs was studied in detail^11,33,34^. The polycondensation reaction between CA and EDA leads to the formation of CA-EDA CPDs. At a low reaction temperature (150 °C), polymer clusters with low molecular weights are formed (Fig. 1b). At elevated temperatures, slightly carbonized polymer clusters (at 200 °C) or carbonized carbon cores (at 300 °C) are formed. The carbon cores in CA-EDA CPDs emerge with increasing temperature. This core formation results in increased photostability and enhanced excitation-dependent PL. However, under these conditions, the QY decreases. This is because the PL center (the molecular state in the crosslinked polymer clusters) exhibits strong PL emissions. The PL center originating from the carbon core exhibits weak PL with high photostability^33^. These carbonization-induced distinct features are common in CA and other amine-containing coreactant-derived CPDs^35–37^.

In other CPD systems, the polymerization–carbonization process can potentially exert varying effects on the CPD structures and their optical properties^25,27,38^. LTrp-LSer CPDs prepared from L-serine (LSer) and L-tryptophan (LTrp)^27^ have taken the form of annular polymer dots at 100 °C, spherical particles with amorphous carbon core structures at 200 °C, and graphitic carbogenic particles at 300 °C. The morphology change is attributed to the enhanced degree of carbonization. An increase in the degree of carbonization results in a decrease in the excitation-dependent PL and an increase in QY (Fig. 1c). Similar observations were not made when CA-EDA CPDs were studied. It was observed that an increase in the carbonization temperature-induced room-temperature ferromagnetism in the LTrp-LSer CPDs (which exhibit strong PL emission) prepared at 300 °C. The calculated and experimentally obtained results revealed that the strong PL emission and ferromagnetism in the highly carbonized LTrp-LSer CPDs could be attributed to the graphitic nitrogen and high crystallinity of the samples. Recently, Lin et al. reported that an increased degree of carbonization could induce ultralong room-temperature phosphorescence (URTP) in phosphoric acid (PA) and EDA-derived CPD systems (Fig. 1d)^38^. The PA-EDA CPDs synthesized at 180 °C exhibited blue fluorescence in water. However, the fluorescence was quenched in the solid state. When the PA-EDA CPDs were further annealed at 280 °C, the dehydration and carbonization processes resulted in the formation of significantly compact cores from the intertwined polymer chains. These compact cores could act as matrices for the immobilization of excited triplet species (N- and P-based groups derived from PA and EDA). These cores could potentially induce the emission of green URTP. The polymerization–carbonization process was found to exert different levels of influence on the structure and properties of the CPD systems based on different precursors, indicating precursor-induced structural diversity in CPD materials.

The CPD structure can be tuned in two ways. In a specific CPD system, the structure can be tuned by regulating the polymerization–carbonization reaction conditions (e.g., reaction temperature, reaction solvent^39^, reaction time, and the ratio of the precursors used^19,40–43^). This inherent structural adjustability (tunable molecular weight or degree of crosslinking in a polymer) is a common feature of polymer-like materials. These methods can potentially be used for tuning the degree of carbonization and optical properties of CPDs to some degree. In addition, the structural features of the precursors can be tuned to achieve structural diversity in the CPDs. The structure of the precursors can also be tuned to synthesize CPDs with varying optical properties and functions. This is a more direct and efficient pathway to fabricate CPDs with ideal properties. Various nonconjugated/conjugated small molecules (bearing different numbers and types of functional groups at different positions), polymers, metal chelates, and biomass materials can be used as precursors. Thus, researchers can tune the properties of the surface groups, molecular states, crosslinked structures, sp^2^ subdomains, and chiral/defect/doping structures^16^. The property of structural tunability is one of the most fascinating features of CPD-based materials.

## Precursor-dependent structural diversity in CPDs

**Fig. 2:**
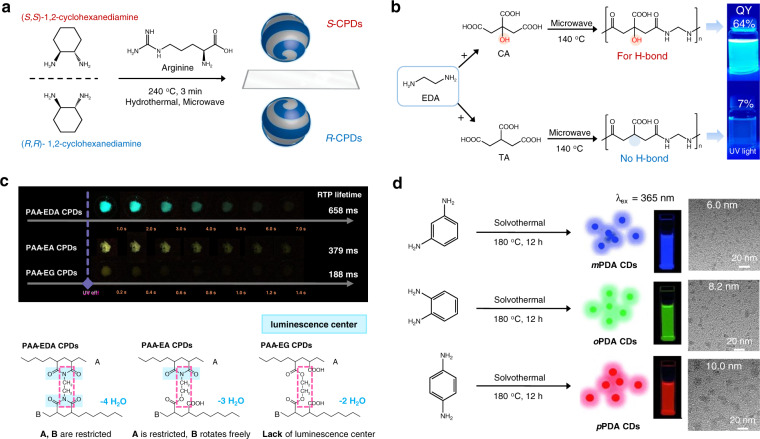
Precursor-dependent emission. **a** Example of the direct synthesis of chiral CPDs from chiral diamine precursors. **b** Reaction of EDA with CA and TA to form CA-EDA CPDs and TA-EDA CPDs. **c** Precursor-dependent RTP property (top) and schematic representation of crosslinking sites (bottom) present in PAA-EDA, PAA-EA, and PAA-EG CPDs. **d** Preparation of RGB PL CPDs from three different phenylenediamine isomers. Reprinted with permission from the literature reports^13,44,51,52^. Copyright 2019 Nature Publishing Group, 2018 American Chemical Society, 2018 Wiley–VCH, 2015 Wiley–VCH.

Molecules or polymer precursors are used as building blocks that can be polymerized and carbonized to form CPDs. The atomic connectivity in CPDs is thus derived from the precursors. Typically, chiral information can be accurately transferred from the precursors to the CPDs (Fig. 2a)^44^. Prato et al. synthesized chiral CPDs based on arginine (Arg) and (*R,R*)- or (*S,S*)-1,2-cyclohexanediamine (CHDA) by using a microwave-assisted hydrothermal method. The circular dichroism (CD) spectral profiles of Arg-RCHDA CPDs and Arg-SCHDA CPDs showed peaks corresponding to negative and positive Cotton effects, respectively. This observation confirms that it is feasible to directly synthesize chiral CPDs from chiral precursor molecules, indicating the genetic relationship between the CPDs and the precursors. These precursor-dependent features allow the precursor molecules to control the properties of the CPD structures. In this section, we highlight the precursor-dominated structural and functional diversity observed in CPD-based materials.

### Precursor-dependent emission

CPDs are emerging as bright and biocompatible fluorescent nanomaterials. The PL emission of CPDs in the visible and near-infrared regions can be effectively tuned. The complex optical properties of CPDs have been summarized in a few review papers published recently^20,21,45^. It has been reported that the emission originates from four types of PL centers (molecular-state, crosslink-enhanced-emission (CEE)-effect-related state, sp^2^ subdomains (carbon core state), and surface state)^45^. The chemical structures of these PL centers, which determine the emissive properties of CPDs, depend on the molecular structures of the precursors.

The molecular-state luminescence and CEE effect significantly influence the PL emission properties of the slightly carbonized CPDs synthesized by the polymerization–carbonization process. Structural fragments of the precursors are retained in the CPD products. Thus, a variation in the precursor structure directly affects the structure and PL properties of the CPDs. For example, condensation reactions between CA and amine-containing compounds result in the formation of fluorescent molecules during the synthesis of incompletely carbonized CA-based CPDs^33,34,46–49^. Molecular fluorophores can potentially be embedded in a carbon/polymer network to produce building blocks of CPDs^50^. These can function as molecular-state PL emissive centers. As shown in Table 1, the molecular fluorophores exhibit different absorption peak positions, emission wavelengths, QY, and PL lifetimes, depending on the amine precursors used. These differences highlight the influences exerted by the different atomic species (N, O, and S) and substituent groups present in the precursors on the structure and optical properties of CPDs.

**Table 1:** Chemical structure and optical properties of molecular fluorophores in CA-based CPDs.

Precursors		Molecular fluorophore	Absorption peaks	PL peak/QY	PL lifetime	Ref.
			234 nm 365 nm	440 nm/38%	6 ns	^34^
			360 nm	450 nm/75%	15 ns	^46^
			240 nm 350 nm	440 nm/85%	14 ns	^33^
			350 nm	450 nm/90%	14 ns	^47^
			242 nm 347 nm	418 nm/66%	9 ns	^48^
			242 nm 344 nm	418 nm/76%	10 ns	^49^

Precursor-dependent structures and emissive properties have also been observed in CEE effect-related CPDs. The CA-EDA CPDs exhibited a high QY (64%), which was significantly higher than those of tricarballylic acid (TA; 7%) and EDA CPDs, although the structures of the copolymers present in both were similar (Fig. 2b)^51^. The strong PL exhibited by CA-EDA CPDs was attributed to the rigid entanglement of the polymer chains. The hydroxyl-induced supramolecular H-bond interactions, which could promote charge transfer between the entangled polyamide chains (effectively enhancing the PL in polymer-cluster-type CPDs), were responsible for the observed entanglement properties. The bright PL emission could be attributed to the CEE effects. The CEE effects also resulted in the generation of unique room-temperature phosphorescence (RTP), which was significantly influenced by the molecular structure of the precursors. Tao et al. investigated the influences of the precursor structures on the RTP in polyacrylic acid (PAA)-based CPDs^13^. PAA and a series of EDA analogs were used to synthesize different types of CPDs (Fig. 2c). Their group confirmed that the crosslinked amide/imide structures function as luminescent centers, inducing strong RTP in PAA-EDA CPDs. It was also reported that less dense and weak crosslinking resulted in the generation of weak RTP in PAA-EA CPDs. RTP was not observed in PAA-EG CPDs. The analysis of the theoretically obtained results revealed that the coupled amide/imide units resulted in the formation of a narrow energy gap, which facilitated intersystem crossing and the generation of the triplet state^13^. This work highlights the inherent relationship (atomic connectivity) between the target CPDs and the molecular precursors, which directly influence the RTP properties.

The precursor-dependent PL properties of CPDs have also been observed in carbon core state (sp^2^-conjugated domains) and surface-state-determined systems. Because of the complex crosslinking/polymerization–carbonization process used for the synthesis of CPDs, the precise chemical structures of these two PL centers have still not been clearly defined. The two PL mechanisms often coexist in a single CPD system. Lin and coworkers reported the preparation of multicolor PL CPDs exhibiting the emission of red, green, and blue PL using three different phenylenediamine isomers (Fig. 2d)^52^. The absorption spectra of *m*-phenylenediamine (mPDA), *o*-phenylenediamine (oPDA), and *p*-phenylenediamine (pPDA) CPDs gradually redshift. The peaks corresponding to the PL of the three CPDs appear at 435, 535, and 604 nm. Increases in the number of sp^2^-conjugated domains and in the nitrogen-doped content in the related products were observed with the change in the position of the amino group (the least in mPDA and the maximum in pPDA). This increase induced the generation of redshifted optical absorption and emission properties^52,53^.

The precursors are the building blocks of CPDs, and their atomic arrangement and reaction behavior influence the polymerization–carbonization process, which influences the chemical structure of the CPD materials. Thus, the chemical structures and optical properties of CPDs can be tuned by tuning the structural properties of the precursors. Environmentally friendly fluorescent CPD materials have been widely used for the development of various optoelectronic devices, bioimaging markers, anti-counterfeit labels, and PL sensors.

### Precursor-dominated versatile functions

**Fig. 3:**
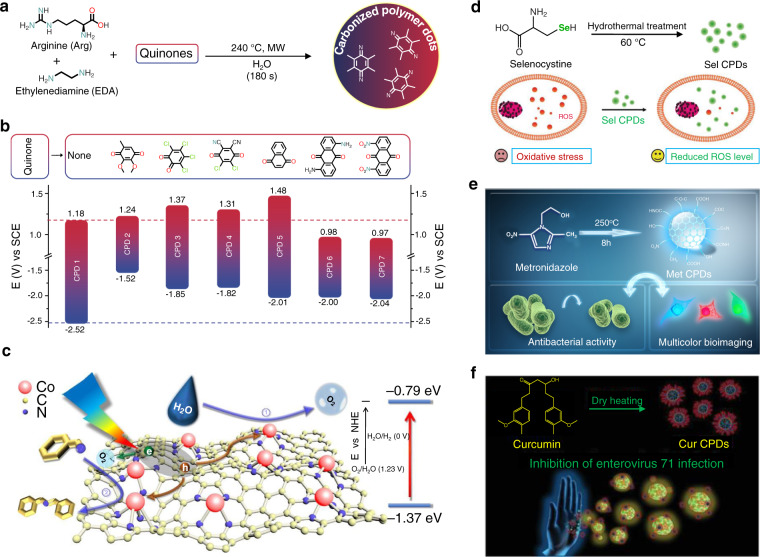
Precursor-dominated versatile functions. **a** Synthesis of CPDs from Arg, EDA, and quinones. **b** Corresponding redox potentials of CPDs. **c** Single Co atom anchored in CPDs derived from VB12 for photocatalysis. **d** Sel-CPDs (exhibiting green fluorescence) synthesized by hydrothermally treating selenocystine, which can be used as an ROS scavenger. **e** Met CPDs exhibiting blue fluorescence and selective antibacterial activity against Porphyromonas gingivalis. **f** Curcumin CPDs and their use as antiviral agents. Reprinted with permission from literature reports^16,56–59^. Copyright 2018, Wiley–VCH, 2020 American Chemical Society, 2018 Wiley–VCH, 2017 Royal Society of Chemistry, 2019 Wiley–VCH.

CPDs exhibit PL-related properties and have a wide range of applications. CPDs have versatile functions depending on the design of precursors. CPDs can also be used in the field of catalysis^54^. They can be used for magnetic resonance imaging^55^ and scavenging free radicals^56^. They can also exhibit antibacterial^57^ and antiviral properties^58^. CPDs have recently shown promise as green photoelectrocatalysts because of their tunable chemical structures and energy levels. Prato et al. prepared a redox library of CPDs using electroactive quinines as co-reactive precursors during the microwave-assisted synthesis of Arg-EDA-based CPDs (Fig. 3a)^16^. As shown in Fig. 3b, the redox potentials of the CPDs can be effectively controlled by tuning the molecular structure of the quinines. This feature makes the CPDs potential photocatalysts. Zhao et al. recently demonstrated that the energy levels in CPD materials influence the photocatalytic performances of lead-halide perovskite/CPD hybrids^17^. They found that the energy level of CPDs prepared from *p*-aminosalicylic acid (*p*ASA) and CA matched well with the energy level of MAPbI_3_. Thus, *p*ASA-CA CPDs can be used to enhance the charge lifetime of MAPbI_3_ perovskites through the process of ultrafast hole extraction. A 35-fold enhancement in the reaction rate (compared to the rate observed when pure MAPbI_3_ was used) of visible-light-driven photocatalytic H_2_ evolution could be achieved. In addition to the electrochemical energy level, the properties of the catalytic active sites of CPDs can be tuned by rationally designing the precursors. Li and coworkers synthesized single atomically anchored cobalt (Co) (on CPDs) via a facile pyrolysis method using a metal chelate of vitamin B12 as the precursor. The single Co atom was stabilized by a defined Co−N_4_ structure inherited from VB12 (Fig. 3c)^59^. The visible-light-driven oxidation reactions could be effectively catalyzed by CPD-supported Co single-atom materials. This work highlights that CPDs can provide a platform for the development of single-atom catalysis.

Luminescent CPDs exhibiting varying functions can be used to fabricate novel multifunctional materials by introducing specific heteroatoms or functional groups into the scaffolds. Zboril et al. reported that Gd-doped CPDs exhibited magnetic resonance imaging properties^55^, while Huang et al. reported that Tb-doped CPDs could selectively detect 2,4,6-trinitrophenol (TNT)^60^. Xu et al. reported that selenium atoms endow selenocysteine-derived CPDs (Ser CPDs) with redox-dependent reversible fluorescence properties. Ser CPDs also exhibited the ability to scavenge free radicals, thus protecting the biosystem from the damage caused by an excess of reactive oxygen species (Fig. 3d)^56^. Liu et al. observed that metronidazole-derived CPDs (Met CPDs) emitted strong PL (blue). The Met CPDs also exhibited higher water solubility than metronidazole. The antibacterial activity exhibited by the Met CPDs against Porphyromonas gingivalis was similar to that exhibited by metronidazole (Fig. 3e)^57^. The antibacterial activity could be attributed to the nitro groups (inherited from the metronidazole precursor) in the Met CPDs. The therapeutic properties of drug CPDs were different from those of the precursors. Curcumin (Cur) exhibits antimicrobial, anticancer, anti-inflammatory, and antioxidant properties, whereas Cur CPDs were found to be effective antiviral agents against enterovirus 71 (EV71). It was believed that the antiviral property originated from the newly formed pyrolytic curcumin polymers (Fig. 3f)^58^. CPD materials exhibiting various novel functions can be fabricated by selecting the appropriate precursors. CPD materials can be used as lubricants^61^, anticorrosive agents^62^, and antioxidants^63^. They can also be used for the upregulation of glycolysis^64^.

The abovementioned examples indicate that structural information can be transferred from precursors to the CPDs via the polymerization–carbonization reaction. The synthesis can more systematically and logically control the structures of CPDs during the polymerization–carbonization process. Given that a large library of CPDs is being created, there is no nomenclature to index the library of CPD materials. This motivates us to establish a system of nomenclature for labeling CPDs. Such a system for deciding the nomenclature of materials can help manage this wide and diverse material system.

## Nomenclature of CPDs

CPDs formed via the polymerization–carbonization process (analogous to polymer materials) are structurally diverse and exhibit the property of polydispersity. The structural features vary within a sample set. Therefore, CPD is better identified as a material and not a precise molecular or nanoscale entity^21^. This is also one of the reasons why most CPD materials exhibit excitation-dependent PL properties. Given the structural features of CPD materials and the structural relationship between CPDs and precursors, the material system can be indexed according to the precursors. Information on the local micro–nanostructure, properties, functions, and application directions of CPDs can be determined from the CPD names. The development of a system to name the CPDs can help classify and index this large family. CPDs will emerge as valuable functional materials in the future because they exhibit low toxicity and excellent optical properties. We believe that the use of a standardized system of nomenclature can be applied in various fields, such as manufacturing and trade.

**Fig. 4:**
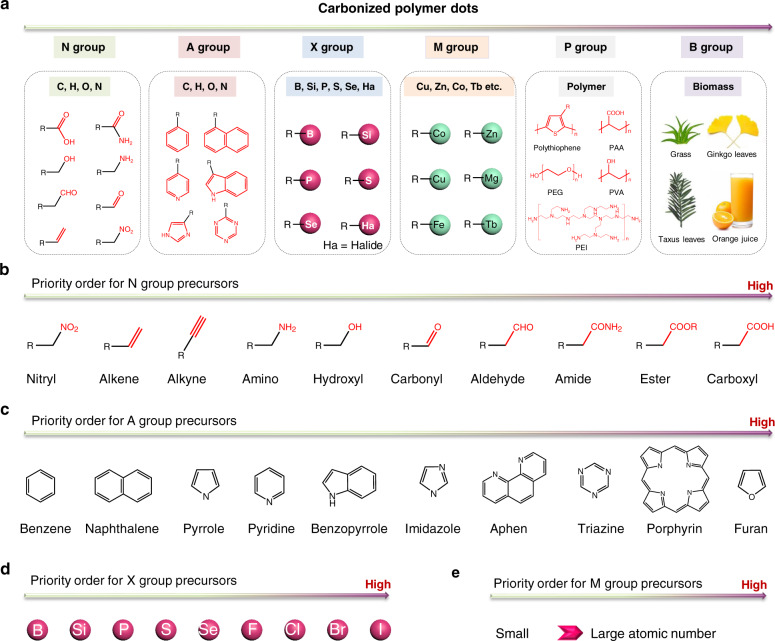
Classification and nomenclature of CPDs. **a** Classification of CPDs based on the precursor structure and elemental composition. **b** Priority order of the nonconjugated precursors based on the functional groups. **c** Priority order of the aromatic precursors based on the conjugated rings. **d** Priority order of the nonmetal-doped precursors. **e** Priority order of the metal-containing precursors.

To better establish the nomenclature, CPDs are divided into six groups based on their elemental composition and chemical structure: N (nonaromatic), A (aromatic), X (nonmetal-doped), M (metal-doped), P (polymer), and B (biomass) groups (Fig. 4a). The elemental composition of the members of groups N and A CPDs is the same (containing C, H, O, or N). Group N consists of CPDs prepared from nonaromatic (aliphatic) small molecules, such as EDA, CA, EA, and acrylamide. Group A consists of CPDs prepared from aromatic molecules and a mixture of aromatic molecules and nonconjugated precursors. Group X consists of CPDs containing nonmetallic elements other than C, H, O, and N (such as B, Si, S, Se, P, and/or halides). Similarly, groups M, P, and B represent metal-doped CPDs, polymer-derived CPDs, and biomass-based CPDs, respectively.

CPDs are generally synthesized from multiple (one to three) molecular precursors. When multiple precursors participate in the formation of CPDs, the precursor names should be hyphenated (e.g., citric-acid-ethanediamine CPDs). The priority order of the precursors should be determined for the nomenclature of each CPD material. Based on the chemical structure of the precursors, the following seven rules have been proposed to determine the priority order of the precursors:

1. Biomass (B) < polymer (P) < metal-doped (M) < nonmetal-doped (X) < aromatic (A) < nonaromatic (N).
  This order can be implemented to identify the priority order of different types of precursors used. For example, CPDs prepared from nonaromatic CA (N-group precursor) and aromatic *p*ASA (A-group precursor) should be identified as *p*ASA-CA CPDs and not as CA-*p*ASA CPDs. In the shorter version of the name, the hyphen (-) should be used to connect each precursor. For another example, PAA and EDA-derived CPDs should be identified as PAA-EDA CPDs.
2. Carboxyl (−COOH) < ester (−COOR) < amide (−CONH−) < aldehyde (−CHO) < carbonyl (−C=O) < hydroxyl (−OH) < amino (−NH_2_) < alkyne (−C≡C) < alkene (−C=C) < nitryl (−NO_2_)
  (I) For naming the N-group CPDs, the priority order of the nonaromatic precursors is determined on the basis of the functional groups present in the scaffold (Fig. 4b). This order is similar to the order followed for naming organic compounds. For example, CA is higher on the priority list than EDA. Thus, the corresponding CPDs should be identified as CA-EDA CPDs.
  (II) When precursors bear the same highest-priority functional groups, the precursors with a greater number of highest-priority groups should be listed first. For example, CPDs prepared from ethylenediaminetetraacetic acid (EDTA; 4 carboxyl groups) and ammonium citrate (AC; 3 carboxyl groups) should be identified as EDTA-AC CPDs.
  (III) When precursors bear the same type and the same number of highest-priority functional groups, the priority order of other functional groups should be considered while naming the CPDs.
3. Furan < porphyrin < triazine < aphen < imidazole < benzopyrrole < pyridine < pyrrole < naphthalene < benzene
  (I) When precursors are aromatic in nature, the priority order is first determined by studying the conjugated structure. There are four rules to follow:
  (i) The *larger the atomic number of heteroatoms* in the aromatic cycle, the higher the priority. For example, furan (O atom) < pyrrole (N atom) < benzene (C atom).
  (ii) The *greater the number of heteroatoms*, the higher the priority. For example, porphyrin < triazine < aphen < benzopyrrole.
  (iii) The *greater the number of aromatic cycles*, the higher the priority. For example, anthracene < naphthalene < benzene.
  (iv) The *larger the aromatic ring* size is, the higher the priority. For example, pyridine < pyrrole.
  Based on the above four rules, the priority order of precursors with common conjugated units is summarized in Rule 3 (Fig. 4c). For example, CPDs derived from L-tryptophan (LTry) and oPDA would be named LTry-oPDA CPDs.
  When the conjugated ring structures of the precursors are the same, the priority order of the precursors is determined on the basis of the substituent groups present (Rule 2). For example, CPDs prepared from phthalic acid (PHA) and oPDA should be named PHA-oPDA CPDs.
4. For nonmetal-doped precursors, the priority order is determined based on the heteroatoms present (Fig. 4d): I < Br < Cl < F < Se < S < P < Si < B. When precursors bear the same heteroatoms in their scaffold, the priority order is determined by Rule 3. Rule 3 is followed by Rule 2.
5. For metal-containing precursors, the priority order is determined by the atomic number: the larger the atomic number is, the higher the priority order (Fig. 4e). When precursors bear the same metallic atoms, the priority order is determined by Rule 4, which is followed by Rule 3 (or Rule 2).
6. When multiple polymers are used as precursors, the priority order is determined by studying the structure of the monomer. The five previously outlined rules are followed.
7. Some special rules:
  (I) Monomers are given a higher priority than crosslinking agents when naming CPDs prepared by the hydrothermal–addition–polymerization–carbonization (HAPC) method because they are generally used for the construction of the main structure of CPDs. For example, CPDs prepared from acrylamide (AM; monomer) and N,N’-methylenediacrylamide (MBA; crosslinking agent) should be named AM-MBA CPDs. When various monomers are used, their priority order is determined following the six rules outlined previously.

To easily understand the nomenclature, the names of the abovementioned typical CPDs are summarized in Table 2. In addition, we named most of the previously reported classical CPD materials following the rules of the nomenclature outlined above. The properties, functions, and fields of application of the CPDs are presented in Table S1. It has been observed that the chemical structures of the precursors are relatively simple and are easy to identify. The majority of the CPD materials can be named following Rules 1 and 2. During the preparation of CPD materials via solvothermal routes, organic solvents (e.g., EDA or glycerol^65,66^, which assuredly act as precursors to form CPDs) should appear in the name of CPDs. Organic solvents such as DMF, DMSO, ethanol, acetonitrile, or formamide are solvents that can be activated or decomposed at high temperature and pressure. These activated solvents or their catabolites may be used as potential precursors for the construction of CPDs. However, the reaction processes are not explicit, so the names of the CPDs do not currently reflect these organic solvents used during synthesis.

**Table 2:** Name of typical CPDs based on the outlined nomenclature.

Precursor structure		Priority order	CPD name	short name
		*p*ASA < CA (Rule 1)	p-aminosalicylic acid-citric-acid CPDs	*p*ASA-CA CPDs
		PAA < EDA (Rule 1)	Polyacrylic-acid-ethylenediamine CPDs	PAA-EDA CPDs
		CA < EDA (Rule 2)	Citric-acid-ethanediamine CPDs	CA-EDA CPDs
		EDTA < AC (Rule 2)	Ethylenediaminetetraacetic-acid-ammonium-citrate CPDs	EDTA-AC CPDs
		LTry < oPDA (Rule 3)	L-tryptophan-o-phenylenediamine CPDs	LTry-oPDA CPDs
		PHA < oPDA (Rule 3)	Phthalic-acid-o-phenylenediamine CPDs	PHA-oPDA CPDs
		AM < MBA (Rule 7)	Acrylamide-N, N’-methylenediacrylamide CPDs	AM-MBA CPDs

## Summary and outlook

The structural and optical properties of CPDs throughout the polymerization–carbonization process have been described. The route followed for the synthesis is responsible for the precursor-dependent diversity and the intrinsic polydispersity observed in CPDs. The CPDs thus synthesized are structurally complex, and a single framework cannot describe fabricated CPDs. We have proposed a system of nomenclature to label and name CPDs. This system of nomenclature can be used to conveniently identify different CPD materials. More than 1500 research papers that describe the synthetic routes and list the applications of CPDs are published in a single year. Given the large library of CPDs that are being created and the strong correlations between the precursor structure and the properties of the formed CPDs, our proposed system of nomenclature will enable the community to better index the diversity of the CPD material system and to further understand the materials’ properties and their control factors in real-world applications. Moreover, the functional properties of some CPD materials have shown great core competitiveness. CPD materials can potentially be used to fabricate optoelectronic devices, biomedical instruments, and anticounterfeiting agents. They have also found their applications in the fields of information encryption and catalysis. The names of the CPDs can be used during the process of commercialization.

In addition, it is critical to develop a platform that can make the fabrication of CPDs more reproducible and controllable. At present, most CPD materials are fabricated through condensation-carbonization procedures from molecular precursors bearing carboxyl, amino, or hydroxyl groups. In this pathway, nucleation and growth are not controlled because of the ubiquitously initiated polymerization reactions in the solution. In addition, the condensation reaction is a reversible process due to the occurrence of reverse hydrolysis, which resists the conversion of the precursors into CPDs. The above two reasons generally lead to final CPDs with a wide size distribution and inhomogeneous properties. This process also generates quite a few byproducts (low molecular weight oligomer clusters or aggregation of carbonaceous materials), which limit the yield of the final CPD products. Inspired by the principle of soap-free emulsion polymerization, our group developed a pathway of addition polymerization and carbonization to fabricate CPDs from precursors bearing double bonds (−C=C)^25,26^. In this reaction system, initiators, monomers, and crosslinking agents are the three key elements to form CPDs. Monomers polymerization is first initiated, forming polymer clusters. Therefore, the sites and amount of nucleation are determined by the initiator. The size of the CPDs can be easily tuned by the number of initiators. More importantly, the addition-polymerization–carbonization method does not involve a reverse reaction. In this case, monomers are capable of completely converting into CPDs, which leads to ultrahigh yields of up to 85%. The addition-polymerization–carbonization reaction procedure is a more controllable and promising platform for the fabrication of CPD materials with uniform properties and morphology. Precursor design and elemental doping should be considered to enrich the optical properties and functions of CPDs in future works.

Finally, another challenge is to determine the detailed structure of CPD materials. New characterization techniques need to be developed. A clear idea about CPD structure can potentially help researchers understand the mechanism of PL generation. Further studies will help in revising and perfecting the proposed system of nomenclature. We encourage researchers working with CPDs to help revise and improve the proposed system of nomenclature to stimulate better development of this field. Such work requires a deeper understanding of the formation process of CPDs. The relationship between the chemical structures of the precursors and the CPDs should also be determined. Further studies on the structural diversity and the proposed system of nomenclature can promote the investigation of synthetic routes and assist in analyzing the structure of the materials. In-depth studies can assist in the further development of the field and help widen the application scope of CPD material systems.

## Supplementary information

Precursor-dependent structural diversity in luminescent carbonized polymer dots (CPDs): the nomenclature
