# Supplementary material for: Precursor-dependent structural diversity in luminescent carbonized polymer dots (CPDs): the nomenclature
Source: Light Sci Appl. 2021 Jul 12;10:142. doi: 10.1038/s41377-021-00579-6 (PMC8275661; doi:10.1038/s41377-021-00579-6)
Supplement: Supplementary file 1 — Precursor-dependent structural diversity in luminescent carbonized polymer dots (CPDs): the nomenclature [file 41377_2021_579_MOESM1_ESM.docx]

**Supporting Information**

**Precursor-dependent structural diversity in luminescent carbonized polymer dots (CPDs): the nomenclature**

Qingsen Zeng, Tanglue Feng, Songyuan Tao, Shoujun Zhu and Bai Yang^*^

State Key Laboratory of Supramolecular Structure and Materials, College of Chemistry, Jilin University, Changchun, 130012, China. E-mail: [byangchem@jlu.edu.cn](mailto:byangchem@jlu.edu.cn)

**Table S1** Precursor, name, property, and application of typical CPDs

**N-group CPDs**

| **Precursors’ structure** | **CPD name** | **Short name** | **Synthetic route** | **Main PL peak** | **QY** | **Additional property** | **Applications** | **Ref.** |
| --- | --- | --- | --- | --- | --- | --- | --- | --- |
|  | citric-acid CPDs | CA CPDs | pyrolysis | 460 nm | 8.9% | PL sensing for Cl^–^ | Cl^–^ detection | [^1^](#_ENREF_1) |
|  | ammonium-citrate CPDs | AC CPDs | hydrothermal | 437 nm | 13.5% | PL sensing for pH | pH sensor | [^2^](#_ENREF_2) |
|  | acetone CPDs | CP CPDs | NAOH-assisted polymerization | ~510 nm | – | charge transfer | sodium-ion batteries | [^3^](#_ENREF_3) |
|  | acetaldehyde CPDs | AcH CPDs | NAOH-assisted polymerization | ~550 nm |  | charge transfer | sodium-ion batteries | [^4^](#_ENREF_4) |
|  | ethylene-glycol CPDs | EG CPDs | hydrothermal | – | – | charge transfer | photocatalytic degradation | [^5^](#_ENREF_5) |
|  | alanine CPDs | Ala CPDs | microwave-hydrothermal | ~430 nm | – | lubricity | hydrogel | [^6^](#_ENREF_6) |
|  | 2-amino-2-hydroxymethyl-propane-1,3-diol CPDs | TRIS CPDs | hydrothermal | 430 nm, | 26.0% | ‒ | bioimaging | [^7^](#_ENREF_7) |
|  | ethylenediaminetetraacetic-acid CPDs | EDTA CPDs | hydrothermal | 430 nm | 26.6% | ‒ | bioimaging | [^7^](#_ENREF_7) |
|  | glycine CPDs | Gly CPDs | hydrothermal | 410 nm | 30.6% | ‒ | bioimaging | [^7^](#_ENREF_7) |
|  | cadaverine CPDs | Cad CPDs | hydrothermal | 430 nm, | 5.4% | ‒ | bioimaging | [^7^](#_ENREF_7) |
|  | glucose CPDs | Glu CPDs | microwave-hydrothermal | 473 nm | 7-11% | – | PL LEDs | [^8^](#_ENREF_8) |
|  | sucrose CPDs | Suc CPDs | hydrothermal | ‒ | ‒ | charge transfer | photocatalysis | [^9^](#_ENREF_9) |
|    | citric-acid-ammonium-hydroxide CPDs | CA-AH CPDs | hydrothermal | ~425 nm | ‒ | up-conversion PL, electron transfer | photoreduction of Cr^6+^ | [^10^](#_ENREF_10) |
|    | citric-acid-urea CPDs | CA-urea CPDs | microwave-hydrothermal | 540 nm | 14% | – | fluorescent Ink | [^11^](#_ENREF_11) |
|    | citric-acid-ethanolamine CPDs | CA-EA CPDs | pyrolysis | 455 nm | 50% | – | – | [^12^](#_ENREF_12) |
|  | citric-acid-2-(2-aminoethoxy)-ethanol CPDs | CA-AEEA CPDs | pyrolysis | 520-650 nm | 3% | – | – | [^13^](#_ENREF_13) |
|    | citric-acid-ethylenediamine CPDs | CA-EDA CPDs | hydrothermal | 443 nm | 80% | PL sensing for Fe^3+^ | multicolor patterning,  Fe^3+^ sensors, bioimaging | [^14^](#_ENREF_14) |
|  | citric-acid-N-ethylethane-1,2-diamine CPDs | CA-EtEDA CPDs | hydrothermal | 450 nm | 77.07% | ‒ | ‒ | [^15^](#_ENREF_15) |
|  | citric-acid-N-(2-aminoethyl)-acetamide CPDs | CA-AEA CPDs | hydrothermal | ‒ | 46.36% | ‒ | ‒ | [^15^](#_ENREF_15) |
|  | citric-acid-diethylenetriamine CPDs | CA-DETA CPDs | pyrolysis | 458 nm | 25.5% | ‒ | imaging-guided drug delivery | [^16^](#_ENREF_16) |
|   | citric-acid-glycine CPDs | CA-Gly CPDs | hydrothermal | 415nm | 16.9% | PL sensing for pH | ‒ | [^17^](#_ENREF_17) |
|    | citric-acid-octadecylamine CPDs | CA-OCTA CPDs | pyrolysis | 450‒600 nm | – | – | – | [^13^](#_ENREF_13) |
|    | citric-acid-1-hexadecylamine CPDs | CA-HDAA CPDs | solvothermal (octadecene) | 420 nm | 40% | ‒ | electroluminescence LEDs | [^18^](#_ENREF_18) |
|    | citric-acid-dicyandiamide CPDs | CA-DCD CPDs | hydrothermal | 452 nm | 36.5% | PL sensing for pH | pH sensor | [^19^](#_ENREF_19) |
|    | citric-acid-tris(hydroxymethyl)aminomethane CPDs | CA-THMA CPDs | pyrolysis | 425 nm | 59.2% | PL sensing for TNP | 2,4,6-trinitrophenol (TNT) detection | [^20^](#_ENREF_20) |
|    | citric-acid-hexamethylenetetramine CPDs | CA-HMTA CPDs | hydrothermal | 420 nm | 17% | ‒ | ‒ | [^21^](#_ENREF_21) |
|    | citric-acid-triethanolamine CPDs | CA-TEA CPDs | hydrothermal | 430 nm | 7% | ‒ | ‒ | [^21^](#_ENREF_21) |
|    | L-ascorbic-acid- ethylene-glycol CPDs | AA-EG CPDs | hydrothermal | ‒ | ‒ | charge transfer | photocatalytic NO_x_ removal | [^22^](#_ENREF_22) |
|  | glycyrrhizic-acid CPDs | GA CPDs | hydrothermal | 438 nm | 1.4% | antiviral activity, scavenging free radicals | antiviral activity | [^23^](#_ENREF_23) |
|    | L-aspartic-acid-D-glucose CPDs | LAsp-Glu CPDs | hydrothermal | 560 nm | ‒ | ‒ | bioimaging | [^24^](#_ENREF_24) |
|    | ascorbic-acid-ethanolamine CPDs | AA-EA CPDs | hydrothermal | ‒ | ‒ | photocatalysis | photocatalytic degradation | [^25^](#_ENREF_25) |
|  | maleic-acid-ethylenediamine CPDs | MA-EDA CPDs | microwave-hydrothermal | 460 nm | 18.9% | solid-state PL | LED phosphors | [^26^](#_ENREF_26) |
|  | oleic-acid-sucrose CPDs | OA-Suc CPDs | pyrolysis | 461nm | 21.6% | ‒ | bioimaging | [^27^](#_ENREF_27) |
|    | glucose-ammonium-hydroxide CPDs | Glu-AH CPDs | hydrothermal | 450-582 nm | 6.8-11.3% | ‒ | photodetector | [^28^](#_ENREF_28) |
|    | methacrylic-acid-*n*-butylamine CPDs | MAA-BA CPDs | hydrothermal | ‒ | ‒ | anticorrosion | corrosion inhibitor for Q235 steel | [^29^](#_ENREF_29) |
|    | arginine-(*S,S*)-1,2-cyclohexanediamine CPDs | Arg-SCHDA CPDs | microwave -hydrothermal | 425 nm | 20% | chiral optical property | chiral supramolecular  porphyrin assemblies | [^30^](#_ENREF_30) |
|    | Arginine-(*R,R*)-1,2-cyclohexanediamine CPDs | Arg-RCHDA CPDs | microwave -hydrothermal | 425 nm | 20% | chiral optical property | chiral supramolecular  porphyrin assemblies | [^30^](#_ENREF_30) |
|      | citric-acid-nitric-acid-ethylenediamine CPDs | CA-NA-EDA CPDs | hydrothermal | ‒ | ‒ | charge transfer | magnetic separation | [^31^](#_ENREF_31) |
|      | citric acid-glycerol- 4,7,10-trioxa-1,13-tridecanediamne CPDs | CA-Gly-TTDDA CPDs | pyrolysis | 437nm | ‒ | ionic sensing | Cu^2+^ detection | [^32^](#_ENREF_32) |

**A-group CPDs**

| **Precursors’ structure** | **CPD name** | **Short name** | **Synthetic route** | **Main PL peak** | **QY** | **Additional property** | **Applications** | **Ref.** |
| --- | --- | --- | --- | --- | --- | --- | --- | --- |
|  | m-phenylenediamine CPDs | mPDA CPDs | solvothermal (ethanol) | 435nm | 17.6% | ‒ | bioimaging | [^33^](#_ENREF_33) |
|  | o-phenylenediamine CPDs | oPDA CPDs | solvothermal (ethanol) | 535nm | 4.8% | ‒ | bioimaging | [^33^](#_ENREF_33) |
|  | p-phenylenediamine CPDs | pPDA CPDs | solvothermal (ethanol) | 604nm | 26.1% | ‒ | bioimaging | [^33^](#_ENREF_33) |
|  | 1,2,4-triamino-benzene CPDs | TAB CPDs | solvothermal (ethanol) | 568nm | 32.5% | PL sensing for ionic and molecular | sensors | [^34^](#_ENREF_34) |
|  | 3-(3,4-dihydroxyphenyl)-L-alanine CPDs | DOPA CPDs, | pyrolysis | 475-555 nm, | ‒ | up-converted PL | bioimaging | [^35^](#_ENREF_35) |
|  | dopamine CPDs | DPA CPDs | hydrothermal | 400 nm | 6.4% | PL sensing for Fe^3+^ and dopamine | Fe^3+^ and dopamine detection, bioimaging | [^36^](#_ENREF_36) |
|  | m-aminobenzoic-acid CPDs | MABA CPDs | hydrothermal | 415 nm | 30.7% | ionic sensing | biosensors | [^37^](#_ENREF_37) |
|  | p-aminosalicylic-acid CPDs | *p*ASA CPDs | solvothermal  (ethanol) | 510 nm | 16.4% | ionic sensing | sensors | [^38^](#_ENREF_38) |
|  | metronidazole CPDs | Met CPDs | hydrothermal | 443 nm | 28.1% | antibacterial activity | antibacterial activity | [^39^](#_ENREF_39) |
|  | folic-acid CPDs | FA-CPDs | hydrothermal | 400 nm | 94.5% | folate receptor | cancer cell-targeted imaging | [^40^](#_ENREF_40) |
|    | p-phenylenediamine-urea CPDs | PPDA-urea CPDs | hydrothermal | 440-625 nm | 35% | ‒ | bioimaging | [^41^](#_ENREF_41) |
|    | *o*-phenylenediamine-nitric-acid CPDs | *o*PDA-NA CPDs | hydrothermal | 630 and 677 nm | 31% | ‒ | bioimaging | [^42^](#_ENREF_42) |
|  | *o*-phenylenediamine-L-glutamic-acid CPDs | *o*PDA-LGluCPDs | hydrothermal &  solvothermal (formamide, DMF, ethanol) | 443-745 nm | 43-54% | ‒ | bioimaging | [^43^](#_ENREF_43) |
|    | *o*-phenylenediamines-ethylenediamine CPDs | *o*PDA-EDACPDs | solvothermal (ethanol) | 550 nm | ‒ | RTP | anticounterfeiting | [^44^](#_ENREF_44) |
|    | catechol-ethanediamine CPDs | Cat-EDA CPDs | solvothermal (ethanediamine) | 510 nm | 49% | molecular sensing | sensors | [^45^](#_ENREF_45) |
|  | dopamine-o-phenylenediamine CPDs | DPA-oPDA CPDs | hydrothermal | 710 nm | 26.3% | two-photon PL | LEDs, bioimaging | [^46^](#_ENREF_46) |
|    | phthalic-acid-o-phenylenediamine CPDs | PHA-oPDA CPDs | solvothermal  (DMF) | 557 nm | 23.7% | two-photon PL | ‒ | [^47^](#_ENREF_47) |
|    | Hydroquinone-ethanediamine CPDs | HQ-EDA-CPDs | hydrothermal | ~520 nm | 45% | two-photon PL | tumor marker detection, drug loading and delivery | [^48^](#_ENREF_48) |
|  | p-aminosalicylic acid-citric-acid CPDs | *p*ASA-CA CPDs | hydrothermal | dual-peak PL  (452, 525 nm) | 49.8% | solid-state PL | LEDs | [^49^](#_ENREF_49) |
|   | *L*-tryptophan-*L*-serine CPDs | LTrp-LSer-CPDs | hydrothermal | 434 nm | 89.6% | ferromagnetism | ‒ | [^50^](#_ENREF_50) |
|  | 2,3-diaminonaphthalen-citric-acid CPDs | DAN-CA CPDs | solvothermal  (ethanol) | 430 nm,  513 nm | 75%  73% | ‒ | electroluminescence LEDs | [^51^](#_ENREF_51) |
|  | 1,5-diaminonaphthalene-citric-acid CPDs | DAN-CA CPDs | solvothermal  (ethanol) | 535 nm,  565 nm  604 nm | 58%  53%  12% |  | electroluminescence LEDs | [^51^](#_ENREF_51) |
|  | pyrene-nitric-acid CPDs | Pyr-NA CPDs | hydrothermal | 525 nm | ‒ | electron transfer | photocatalytic RhB degradation | [^52^](#_ENREF_52) |
|  | folic-acid-ethylene-glycol CPDs | FA-EG CPDs | hydrothermal | 390 nm | 15.7% | PL sensing for Hg^2+^ | Hg^2+^ detection | [^53^](#_ENREF_53) |
|  | 1,3,6-trinitropyrene-pyrene-nitric-acid CPDs | TNP-Pyr-NA CPDs | hydrothermal | ‒ | ‒ | photocatalysis | photocatalytic H_2_ evolution | [^54^](#_ENREF_54) |

**X-group CPDs**

| **Precursors’ structure** | **CPD name** | **Short name** | **Synthetic route** | **Main PL peak** | **QY** | **Additional property** | **Applications** | **Ref.** |
| --- | --- | --- | --- | --- | --- | --- | --- | --- |
|  | 3-aminobenzeneboronic-acid CPDs | ABBA CPDs | hydrothermal | 504 nm | ‒ | energy transfer | phosphorus pesticide detection | [^55^](#_ENREF_55) |
|  | phenylboronic-acid CPDs | PBA CPDs | hydrothermal | 408 nm | 8.4% | PL sensing for glucose | glucose sensor | [^56^](#_ENREF_56) |
|  | N-[3-(trimethoxysilyl)propyl]ethylenediamine-glycerol CPDs | DAMO-Gly CPDs | solvothermal (glycerol) | 442 nm | 45% | on-off-on of PL | bioimaging | [^57^](#_ENREF_57) |
|  | N-(*β*-aminoethyl)-*γ*-aminopropyl methyldimethoxy silane-citric-acid CPDs | AEAPMS-CA CPDs | pyrolysis | 450 nm | 47% | ‒ | bioimaging | [^58^](#_ENREF_58) |
|  | N-[3-(trimethoxysilyl)propyl]-  ethylenediamine-catechol CPDs | DAMO-Cat CPDs | hydrothermal | 512nm | ‒ | molecular sensing | sensors | [^59^](#_ENREF_59) |
|  | adenosine-5’-triphosphate CPDs | ATP CPDs | hydrothermal | 408nm | 43.2% | ionic sensing | Fe^3+^ nanoprobe | [^60^](#_ENREF_60) |
|  | [phosphoric](C:/%E8%BD%AF%E4%BB%B6/after%20install/after%20install/8.9.5.0/resultui/html/index.html#/javascript:;)-[acid](C:/%E8%BD%AF%E4%BB%B6/after%20install/after%20install/8.9.5.0/resultui/html/index.html#/javascript:;)-ethylenediamine CPDs | PA-EDA CPDs | hydrothermal | 413 nm | 21.8% in H_2_O  5.3% in powder | RTP (538 nm) | anticounterfeiting | [^61^](#_ENREF_61) |
|  | phosphoric-acid-ethanolamine CPDs | PA-EA CPDs | microwave | 417 nm | 20.5% in H_2_O  3.5% in powder | RTP (535 nm) | anti-counterfeiting, information protection | [^62^](#_ENREF_62) |
|  | phosphoric-acid-sucrose CPDs | PA- Suc CPDs | microwave-hydrothermal | 453 nm | ‒ | charge transfer | bioimaging | [^63^](#_ENREF_63) |
|  | phosphoric-acid-glucose-ethylendiamine CPDs | PA-Glu-EDA  CPDs | pyrolysis | 502nm | ‒ | ionic sensing | sensors  bioimaging | [^64^](#_ENREF_64) |
|  | phosphoric-acid-glucose-ammonia CPDs | PA-Glu-AH  CPDs | hydrothermal | 437nm | 54% | ionic sensing | Fe^3+^ detection  and bioimaging | [^65^](#_ENREF_65) |
|  | ammonium-phosphate-4,7,10-trioxa-1,13-  tridecanediamine-glycerol CPDs | AP-Gly-TTDDA CPDs | microwave-  hydrothermal | ‒ | ‒ | photocatalysis | photocatalytic NO oxidation | [^66^](#_ENREF_66) |
|  | *L*-cysteine CPDs | LCys CPDs | hydrothermal | 510 nm | 41.3% | chiral optical properties, up-regulated glycolysis | regulation on cellular energy metabolism | [^67^](#_ENREF_67) |
|  | sulphuric-acid-fructose CPDs | SA-Fru CPDs | hydrothermal | 617 nm | 7.1% | ‒ | ‒ | [^68^](#_ENREF_68) |
|  | 3-mercaptopropionic-acid-1,3,6-trinitropyrene CPDs | MPA-TNP  CPDs | hydrothermal | 450nm | 9.2% | PL sensing for ions | Ag^+^ detection | [^69^](#_ENREF_69) |
|  | L-cysteine-citric-acid CPDs | Cys-CA CPDs | hydrothermal | 415nm, | 73% | PL sensing for pH | ‒ | [^17^](#_ENREF_17) |
|  | thiourea-citric-acid CPDs | TU-CA CPDs | hydrothermal | 450nm | 71%， | photocatalysis | photocatalytic degradation | [^70^](#_ENREF_70) |
| 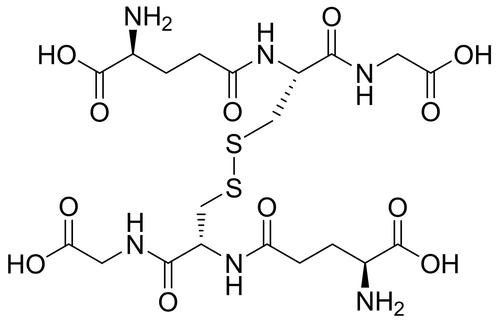 | glutathione-glucose CPDs | GSH-Glu CPDs | hydrothermal | 471 nm | 7.2% | PL sensing for pH and temperature | ‒ | [^71^](#_ENREF_71) |
|  | L-cystine-*o*-phenylenediamine CPDs | LCys-*o*PDA CPDs | solvothermal (ethanol) | 595 and 648 nm | 35.7% | PL sensing for pH | cell imaging | [^72^](#_ENREF_72) |
|  | 2,5-diaminobenzenesulfonic-acid-4-aminophenylboronic-acid CPDs | DABSA-APB CPDs | hydrothermal | 600nm | 5.4% | ionic sensing | Fe^3+^ detection, bioimaging | [^73^](#_ENREF_73) |
|  | dithiosalicylic-acid-melamine- acetic-acid CPDs | MA-DTSA-HAc CPDs | high-pressure pyrolysis | 467 nm in H_2_O,  620 nm in  powder | 6.0%-H_2_O,  3.5% in powder | aggregation-induced emission | anticounterfeiting, information encryption | [^74^](#_ENREF_74) |
|  | selenocystine CPDs | Sel CPDs | hydrothermal | 490 nm | 7.6% | redox-dependent luminescence | scavenging free radicals | [^75^](#_ENREF_75) |
|  | dexamethasone-1,2,4,5-tetraaminobenzene CPDs | Dex-TAB CPDs | microwave-hydrothermal | 610 nm | ‒ | lysosome-targeting | ratiometric imaging of formaldehyde | [^76^](#_ENREF_76) |
|  | triethylamine-trihydrofluoride-glucose CPDs | TEATHF-Glu CPDs | solvothermal (DMF, acetonitrile) | 435 nm (H_2_O) | 55.7%-H_2_O  12.4%-solid state | RTP-455 nm | anticounterfeiting, pattern steganography | [^77^](#_ENREF_77) |
|  | carbon-tetrachloride-ethylenediamine CPDs | CTC-EDA CPDs | solvothermal | 406 nm | 20.4%, | PL sensing for pH, Ag^+^, Fe^3+^, H_2_O_2_ | Ag^+^, Fe^3+^, H_2_O_2_ detection and bioimaging | [^78^](#_ENREF_78) |
|  | chloroform- ethylenediamine CPDs | CF-DEA CPDs | reflux | 407 nm,  555 nm | 17.1%,  12.6% | PL sensing for pH | pH sensor | [^79^](#_ENREF_79) |
|  | guanidine-hydrochloride-citric-acid CPDs | GACl-CA CPDs | hydrothermal | 460nm | 60.5% | ionic sensing | bioimaging | [^80^](#_ENREF_80) |
|  | neutral-red-citric-acid CPDs | NR-CA CPDs | hydrothermal | 632 nm | 12.1% | PL sensing for metal ions | ion sensing | [^81^](#_ENREF_81) |
|  | basic-fuchsin-citric acid CPDs | BF-CA CPDs | hydrothermal | 475 and 545nm | 54% | ionic sensing | sensors | [^82^](#_ENREF_82) |

**M-group CPDs**

| **Precursors’ structure** | **CPD name** | **Short name** | **Synthetic route** | **Main PL peak** | **QY** | **Additional property** | **Applications** | **Ref.** |
| --- | --- | --- | --- | --- | --- | --- | --- | --- |
|  | sodium-citrate CPDs | NaCA-CPDs | hydrothermal | ~500 nm | ‒ | charge transfer | photocatalytic degradation | [^83^](#_ENREF_83) |
|  | ethylenediaminetetraacetic-acid-disodium-salt CPDs | EDTA2Na CPDs | pyrolysis | 410 nm | 11% | PL sensing for Hg^2+^ and biothiols | Hg^2+^ and biothiols detection | [^84^](#_ENREF_84) |
|  | sodium-citrate-ammonium-hydrogen-carbonate CPDs | NaCA-AHCCPDs | hydrothermal | 435 nm | 68% | PL sensing for Hg^2+^ | Hg^2+^ probing | [^85^](#_ENREF_85) |
|  | sodium-citrate-ethylenediamine CPDs | NaCA-EDACPDs | hydrothermal | ‒ | ‒ | charge transfer | H_2_O_2_ detection | [^86^](#_ENREF_86) |
|  | sodium-thiosulfate-sodium-citrate CPDs | HYPO-NaCA CPDs | hydrothermal | 440nm | 67% | ionic sensing | Fe^3+^ detection | [^87^](#_ENREF_87) |
|  | monopotassium phosphate-glucose CPDs | MKP-Glu CPDs | hydrothermal | 435nm,  510 nm | 2.4%,  1.1% | ‒ | bioimaging | [^88^](#_ENREF_88) |
|  | chlorophyll CPDs | Chl CPDs | hydrothermal | 520nm | ‒ | energy sensing | sensor | [^89^](#_ENREF_89) |
|  | manganese-phthalocyanine CPDs | MnPC CPDs | solvothermal (ethanol) | 745 nm | ‒ | magnetic resonance imaging, catalyzing H_2_O_2_ | simultaneous bimodal imaging and photodynamic therapy | [^90^](#_ENREF_90) |
|  | vitamin-B12 CPDs | VB12 CPDs | pyrolysis | 425 nm |  | photocatalytic activity | photocatalytic H_2_O splitting, organic synthesis, and degradation | [^91^](#_ENREF_91) |
|  | copper-acetate-ascorbic-acid CPDs | AA-CuAc CPDs | hydrothermal | 455 nm | 3.22% | PL sensing for pH, upconverted PL, polarity-dependent PL | ‒ | [^92^](#_ENREF_92) |
|  | zinc-gluconate CPDs | ZnGlu CPDs | pyrolysis | 460 nm | 13.9% | PL sensing for EDTA | EDTA detection | [^88^](#_ENREF_88) |
|  | terbium-nitrate-citric-acid CPDs | TbN-CA CPDs | pyrolysis | 450 nm | ‒ | PL sensing fot pH and 2,4,6-trinitrophenol | 2,4,6-trinitrophenol detection | [^93^](#_ENREF_93) |
|  | gadopentetic acid-betaine hydrochloride-tris(hydroxymethyl)aminomethane CPDs | GdDTPA-BCl- THMA CPDs | pyrolysis | 440 nm | ‒ | magnetic resonance imaging (MRI) | dual fluorescent-MRI probe | [^94^](#_ENREF_94) |

**P-group CPDs**

| **Precursors’ structure** | **CPD name** | **Short name** | **Synthetic route** | **Main PL peak** | **QY** | **Additional property** | **Applications** | **Ref.** |
| --- | --- | --- | --- | --- | --- | --- | --- | --- |
|  | Polyvinyl-alcohol CPDs | PVA  CPDs | hydrothermal | 540 nm | ‒ | solid-state PL | LED | [^95^](#_ENREF_95) |
|  | poly(ethylene glycol) CPDs | PEG CPDs | microwave-hydrothermal | 450 nm | 54% | ‒ | bioimaging | [^96^](#_ENREF_96) |
|  | chitosan CPDs | CS-CPDs | pyrolysis | 407 nm | ‒ | adsorption capacity for metal ions | U(VI) and ^241^Am(III) removal | [^97^](#_ENREF_97) |
|  | chitin CPDs | CT CPDs | solvothermal (ethanol) | 475 nm, | 11.6%, | charge transfer | solar cell | [^98^](#_ENREF_98) |
|  | polyacrylic-acid-ethylenediamine CPDs | PAA-EDA CPDs | hydrothermal | 410 nm in H_2_O  494 nm in  powder | 32.4% in H_2_O  28.8% in powder | RTP | anticounterfeiting | [^99^](#_ENREF_99) |
|  | poly(vinyl alcohol)-ethylenediamine CPDs | PVA-EDA CPDs | hydrothermal | 414 nm | 35% | solid-state PL | LEDs | [^100^](#_ENREF_100) |
|  | polythiophene-phenylpropionic-acid CPDs | PPA CPDs | hydrothermal | 640 nm | 2.3% | ‒ | fluorescence imaging, photoacoustic imaging and photothermal therapy | [^101^](#_ENREF_101) |
|  | poly(N,N-dimethyl-N′-(4-(thiophen-3-yl)benzyl)dodecan-1-aminium bromide) CPDs | PDTBDAB CPDs | hydrothermal | 680 nm | 1.3% | ‒ | bioimaging, photodynamic therapy | [^102^](#_ENREF_102) |
|  | poly(ethylenimine)-citric-acid CPDs | PEI-CA CPDs | pyrolysis | 450 nm | 40% | PL sensing for Cu^2+^ | Cu^2+^ detection | [^103^](#_ENREF_103) |
|  | poly(ethylenimine)-dopamine CPDs | PEI-DPA CPDs | hydrothermal | 526 nm | ‒ | ‒ | bioimaging | [^104^](#_ENREF_104) |
|  | polyoxyethylene-bis(amine)-citric-acid CPDs | PEGBA-CA CPDs | hydrothermal | 435 nm | 31% | PL sensing for Fe^3+^ | Fe^3+^ detection | [^105^](#_ENREF_105) |
|  | poly(ethylenimine)-glycerol CPDs | PEI-Gly CPDs | microwave-pyrolysis | 470 nm，  470 nm，  470 nm | 9.4%,  15.3%,  7.0% | ‒ | bioimaging | [^106^](#_ENREF_106) |
|  | poly(ethyleneimine)-glucose CPDs | PEI-Glu CPDs | hydrothermal | 466 nm, | 2.9% | antibacterial activity | antibacterial activity | [^107^](#_ENREF_107) |
|  | linear-poly(ethyleneimine)-glucose CPDs | LPEI-GLu CPDs | hydrothermal | 473 nm | 2.4% | antibacterial activity | antibacterial activity | [^107^](#_ENREF_107) |
|  | polystyrene-poly(glycidyl methacrylate) CPDs | PS-PGMA CPDs | pyrolysis | 383 nm,  464 nm,  424 nm | 47%,  41%,  25% | ‒ | LEDs | [^108^](#_ENREF_108) |
|  | poly(N,N-dimethyl-N′-(4-(thiophen-3-yl)benzyl)dodecan-1-aminium bromide)- diphenyl-diselenide CPDs | PDTBDAB-DPDS CPDs | hydrothermal | 731 and 820 nm | 0.2% | two-photon PL | bioimaging, photothermal therapy | [^109^](#_ENREF_109) |
|  | chitosan-acetic-acid-EDA CPDs | CS-HAc-EDA CPDs | microwave-pyrolysis | 417 nm | 20.1% | PL sensing for Fe^3+^ | Fe^3+^ probing, bioimaging | [^110^](#_ENREF_110) |
|  | poly(ethylenimine)-sodium-borohydride-citric-acid CPDs | PEI-NaBH-CA CPDs | hydrothermal | 400 nm,  460 nm,  500 nm | 20%,  15%,  12% | ‒ | ‒ | [^111^](#_ENREF_111) |
|  | poly(ethylenimine)-phosphoric-acid-ethylene-glycol CPDs | PEI-PA-EG CPDs, | hydrothermal | 610 nm,  710 nm, | 7.5%,  6% |  |  | [^111^](#_ENREF_111) |
|  | poly(ethylene glycol)-serine-glycerol CPDs | PEG-Ser-Gly CPDs | microwave-pyrolysis | 440 nm | 12% | PL sensing for nitrite | nitrite detection | [^112^](#_ENREF_112) |
|  | poly(ethylene glycol)-sulfuric-acid-sucrose CPDs | PEG-SA-SucCPDs | microwave | 527 nm | ‒ | ‒ | melamine sensing | [^113^](#_ENREF_113) |

**B-group CPDs**

| **Precursors** | **CPD name** | **Synthetic route** | **Main PL peak** | **QY** | **Additional property** | **Applications** | **Ref.** |
| --- | --- | --- | --- | --- | --- | --- | --- |
| grass | grass CPDs | hydrothermal | 443 nm | 6.2% | Cu^2+^ sensing | Cu^2+^ detection | [^114^](#_ENREF_114) |
| pomelo peel | pomelo-peel CPDs | hydrothermal | 444 nm | 6.9% | Hg^2+^ sensing | Hg^2+^ detection | [^115^](#_ENREF_115) |
| soy milk | soy-milk CPDs | hydrothermal | 426 nm | 2.6% | electron transfer | electrocatalytic O_2_ reduction | [^116^](#_ENREF_116) |
| orange  juice | orange-juice CPDs | hydrothermal | 441 nm | 26% | ‒ | bioimaging | [^117^](#_ENREF_117) |
| coffee grounds | coffee-grounds CPDs | pyrolysis | 440 nm | 3.8% | ‒ | bioimaging & SALDI-MS matrices | [^118^](#_ENREF_118) |
| banana juice | banana-juice CPDs | hydrothermal | 460 nm | 9.0% | pH sensing | ‒ | [^119^](#_ENREF_119) |
| cocoon silk | cocoon-silk CPDs | hydrothermal | 415 nm | 38 % | Hg^2+^  and Fe^3+^ sensing | bioimaging | [^120^](#_ENREF_120) |
| gelatin | gelatin CPDs | hydrothermal | 430 nm | 31.6% | pH sensing,  up-converted PL | bioimaging & PL ink | [^121^](#_ENREF_121) |
| honey | honey CPDs | hydrothermal | 420 nm | 19.8% | up-converted PL,  Fe^3+^ sensing | bioimaging & Fe3+ detection | [^122^](#_ENREF_122) |
| bovine serum albumin | BSA CPDs | solvothermal (ethanol) | 440 nm | 7% | PL sensing for PH | bioimaging & drug delivery | [^123^](#_ENREF_123) |
| milk | milk CPDs | hydrothermal | 454 nm | 12% | ‒ | bioimaging | [^124^](#_ENREF_124) |
| apple juice | apple-juice CPDs | hydrothermal | 475 nm | 4.27% | ‒ | bioimaging | [^125^](#_ENREF_125) |
| aloe | aloe CPDs | hydrothermal | 503 nm | 10.4% | tartrazine sensing | tartrazine detection | [^126^](#_ENREF_126) |
| coriander leaves | coriander-leaves CPDs | hydrothermal | 400 nm | 6.48% | antioxidant activity, Fe^3+^ sensing | antioxidants, Fe^3+^ sensors &  bioimaging | [^127^](#_ENREF_127) |
| papaya | papaya CPDs | hydrothermal | 450 nm | 19.0% | Fe^3+^ sensing | fluorescent sensing | [^128^](#_ENREF_128) |
| prunus avium | prunus-avium CPDs | hydrothermal | 411 nm | 13% | Fe^3+^ sensing | Fe^3+^ detection &  bioimaging | [^129^](#_ENREF_129) |
| phyllanthus acidus | phyllanthus-acidus CPDs | hydrothermal | 420 nm | 14% | Fe^3+^ sensing | Fe^3+^ detection, bio imaging, fluorescent ink | [^130^](#_ENREF_130) |
| ginkgo-leaves | ginkgo-leaves CPDs | hydrothermal | 427 nm | ‒ | electron transfer | electrocatalytic H_2_ evolution | [^131^](#_ENREF_131) |
| garlic | garlic CPDs | hydrothermal | 442 nm | 17.5% | antioxidant activity | free radical scavenging &  bioimaging | [^132^](#_ENREF_132) |
| white pepper | white-pepper CPDs | reflux (ethanol) | 520 and 668 nm | ‒ | PL sensing for metal ions | coenzyme A sensor | [^133^](#_ENREF_133) |
| porphyra | porphyra CPDs | solvothermal (ethanol/water) | 450 nm | ‒ | supporting capacity | electrocatalytic H_2_ evolution | [^134^](#_ENREF_134) |
| rice residue and lysine | rice-residue-lysine CPDs | hydrothermal | 440 nm | 23.5% | ‒ | detecting Fe^3+^ ions and tetracyclines | [^135^](#_ENREF_135) |

**References**

(1) Dong, Y.; Li, G.; Zhou, N.; Wang, R.; Chi, Y.; Chen, G., Graphene quantum dot as a green and facile sensor for free chlorine in drinking water. *Anal. Chem.* **2012**, *84*, 8378-8382.

(2) Yang, Z.; Xu, M.; Liu, Y.; He, F.; Gao, F.; Su, Y.; Wei, H.; Zhang, Y., Nitrogen-doped, carbon-rich, highly photoluminescent carbon dots from ammonium citrate. *Nanoscale* **2014**, *6*, 1890-1895.

(3) Zhang, Y.; Foster, C. W.; Banks, C. E.; Shao, L.; Hou, H.; Zou, G.; Chen, J.; Huang, Z.; Ji, X., Graphene-Rich Wrapped Petal-Like Rutile TiO2tuned by Carbon Dots for High-Performance Sodium Storage. *Adv. Mater.* **2016**, *28*, 9391-9399.

(4) Hou, H.; Shao, L.; Zhang, Y.; Zou, G.; Chen, J.; Ji, X., Large-Area Carbon Nanosheets Doped with Phosphorus: A High-Performance Anode Material for Sodium-Ion Batteries. *Advanced Science* **2017**, *4*, 1600243.

(5) Bilal Tahir, M.; Sagir, M., Carbon nanodots and rare metals (RM = La, Gd, Er) doped tungsten oxide nanostructures for photocatalytic dyes degradation and hydrogen production. *Sep. Purif. Technol.* **2019**, *209*, 94-102.

(6) Guo, J.; Mei, T.; Li, Y.; Hafezi, M.; Lu, H.; Li, J.; Dong, G., One-pot synthesis and lubricity of fluorescent carbon dots applied on PCL-PEG-PCL hydrogel. *Journal of Biomaterials Science, Polymer Edition* **2018**, *29*, 1549-1565.

(7) Hsu, P.-C.; Chang, H.-T., Synthesis of high-quality carbon nanodots from hydrophilic compounds: role of functional groups. *Chem. Commun.* **2012**, *48*, 3984.

(8) Tang, L.; Ji, R.; Cao, X.; Lin, J.; Jiang, H.; Li, X.; Teng, K. S.; Luk, C. M.; Zeng, S.; Hao, J., Deep ultraviolet photoluminescence of water-soluble self-passivated graphene quantum dots. *ACS nano* **2012**, *6*, 5102-5110.

(9) Ye, K.-H.; Wang, Z.; Gu, J.; Xiao, S.; Yuan, Y.; Zhu, Y.; Zhang, Y.; Mai, W.; Yang, S., Carbon quantum dots as a visible light sensitizer to significantly increase the solar water splitting performance of bismuth vanadate photoanodes. *Energy & Environmental Science* **2017**, *10*, 772-779.

(10) Li, Y.; Liu, Z.; Wu, Y.; Chen, J.; Zhao, J.; Jin, F.; Na, P., Carbon dots-TiO2 nanosheets composites for photoreduction of Cr(VI) under sunlight illumination: Favorable role of carbon dots. *Appl. Catal., B* **2018**, *224*, 508-517.

(11) Qu, S.; Wang, X.; Lu, Q.; Liu, X.; Wang, L., A Biocompatible Fluorescent Ink Based on Water-Soluble Luminescent Carbon Nanodots. *Angew. Chem. Int. Ed.* **2012**, *51*, 12215-12218.

(12) Krysmann, M. J.; Kelarakis, A.; Dallas, P.; Giannelis, E. P., Formation Mechanism of Carbogenic Nanoparticles with Dual Photoluminescence Emission. *J. Am. Chem. Soc.* **2011**, *134*, 747-750.

(13) Bourlinos, A. B.; Stassinopoulos, A.; Anglos, D.; Zboril, R.; Karakassides, M.; Giannelis, E. P., Surface Functionalized Carbogenic Quantum Dots. *Small* **2008**, *4*, 455-458.

(14) Zhu, S.; Meng, Q.; Wang, L.; Zhang, J.; Song, Y.; Jin, H.; Zhang, K.; Sun, H.; Wang, H.; Yang, B., Highly Photoluminescent Carbon Dots for Multicolor Patterning, Sensors, and Bioimaging. *Angew. Chem. Int. Ed.* **2013**, *52*, 3953-3957.

(15) Song, Y.; Zhu, S.; Zhang, S.; Fu, Y.; Wang, L.; Zhao, X.; Yang, B., Investigation from chemical structure to photoluminescent mechanism: a type of carbon dots from the pyrolysis of citric acid and an amine. *Journal of Materials Chemistry C* **2015**, *3*, 5976-5984.

(16) Feng, T.; Ai, X.; An, G.; Yang, P.; Zhao, Y., Charge-Convertible Carbon Dots for Imaging-Guided Drug Delivery with Enhanced in Vivo Cancer Therapeutic Efficiency. *ACS Nano* **2016**, *10*, 4410-4420.

(17) Dong, Y.; Pang, H.; Yang, H. B.; Guo, C.; Shao, J.; Chi, Y.; Li, C. M.; Yu, T., Carbon-Based Dots Co-doped with Nitrogen and Sulfur for High Quantum Yield and Excitation-Independent Emission. *Angew. Chem. Int. Ed.* **2013**, *52*, 7800-7804.

(18) Zhang, X.; Zhang, Y.; Wang, Y.; Kalytchuk, S.; Kershaw, S. V.; Wang, Y.; Wang, P.; Zhang, T.; Zhao, Y.; Zhang, H., Color-switchable electroluminescence of carbon dot light-emitting diodes. *ACS nano* **2013**, *7*, 11234-11241.

(19) Wu, Z. L.; Gao, M. X.; Wang, T. T.; Wan, X. Y.; Zheng, L. L.; Huang, C. Z., A general quantitative pH sensor developed with dicyandiamide N-doped high quantum yield graphene quantum dots. *Nanoscale* **2014**, *6*, 3868-3874.

(20) Lin, L.; Rong, M.; Lu, S.; Song, X.; Zhong, Y.; Yan, J.; Wang, Y.; Chen, X., A facile synthesis of highly luminescent nitrogen-doped graphene quantum dots for the detection of 2,4,6-trinitrophenol in aqueous solution. *Nanoscale* **2015**, *7*, 1872-1878.

(21) Schneider, J.; Reckmeier, C. J.; Xiong, Y.; von Seckendorff, M.; Susha, A. S.; Kasák, P.; Rogach, A. L., Molecular Fluorescence in Citric Acid-Based Carbon Dots. *The Journal of Physical Chemistry C* **2017**, *121*, 2014-2022.

(22) Huang, Y.; Gao, Y.; Zhang, Q.; Zhang, Y.; Cao, J.-j.; Ho, W.; Lee, S. C., Biocompatible FeOOH-Carbon quantum dots nanocomposites for gaseous NO removal under visible light: Improved charge separation and High selectivity. *J. Hazard. Mater.* **2018**, *354*, 54-62.

(23) Tong, T.; Hu, H.; Zhou, J.; Deng, S.; Zhang, X.; Tang, W.; Fang, L.; Xiao, S.; Liang, J., Glycyrrhizic‐Acid‐Based Carbon Dots with High Antiviral Activity by Multisite Inhibition Mechanisms. *Small* **2020**, *16*, 1906206.

(24) Zheng, M.; Ruan, S.; Liu, S.; Sun, T.; Qu, D.; Zhao, H.; Xie, Z.; Gao, H.; Jing, X.; Sun, Z., Self-targeting fluorescent carbon dots for diagnosis of brain cancer cells. *ACS nano* **2015**, *9*, 11455-11461.

(25) Song, B.; Wang, T.; Sun, H.; Shao, Q.; Zhao, J.; Song, K.; Hao, L.; Wang, L.; Guo, Z., Two-step hydrothermally synthesized carbon nanodots/WO3 photocatalysts with enhanced photocatalytic performance. *Dalton Transactions* **2017**, *46*, 15769-15777.

(26) Shao, J.; Zhu, S.; Liu, H.; Song, Y.; Tao, S.; Yang, B., Full-Color Emission Polymer Carbon Dots with Quench-Resistant Solid-State Fluorescence. *Advanced Science* **2017**, *4*, 1700395.

(27) Chen, B.; Li, F.; Li, S.; Weng, W.; Guo, H.; Guo, T.; Zhang, X.; Chen, Y.; Huang, T.; Hong, X.; You, S.; Lin, Y.; Zeng, K.; Chen, S., Large scale synthesis of photoluminescent carbon nanodots and their application for bioimaging. *Nanoscale* **2013**, *5*, 1967.

(28) Tang, L.; Ji, R.; Li, X.; Bai, G.; Liu, C. P.; Hao, J.; Lin, J.; Jiang, H.; Teng, K. S.; Yang, Z., Deep ultraviolet to near-infrared emission and photoresponse in layered N-doped graphene quantum dots. *ACS nano* **2014**, *8*, 6312-6320.

(29) Ye, Y.; Zou, Y.; Jiang, Z.; Yang, Q.; Chen, L.; Guo, S.; Chen, H., An effective corrosion inhibitor of N doped carbon dots for Q235 steel in 1 M HCl solution. *J. Alloys Compd.* **2020**, *815*, 152338.

(30) Ðorđević, L.; Arcudi, F.; D’Urso, A.; Cacioppo, M.; Micali, N.; Bürgi, T.; Purrello, R.; Prato, M., Design principles of chiral carbon nanodots help convey chirality from molecular to nanoscale level. *Nat. Commun.* **2018**, *9*.

(31) Deng, Y.; Ok, Y. S.; Mohan, D.; Pittman, C. U.; Dou, X., Carbamazepine removal from water by carbon dot-modified magnetic carbon nanotubes. *Environ. Res.* **2019**, *169*, 434-444.

(32) Wang, Y.; Zhang, C.; Chen, X.; Yang, B.; Yang, L.; Jiang, C.; Zhang, Z., Ratiometric fluorescent paper sensor utilizing hybrid carbon dots–quantum dots for the visual determination of copper ions. *Nanoscale* **2016**, *8*, 5977-5984.

(33) Jiang, K.; Sun, S.; Zhang, L.; Lu, Y.; Wu, A.; Cai, C.; Lin, H., Red, Green, and Blue Luminescence by Carbon Dots: Full-Color Emission Tuning and Multicolor Cellular Imaging. *Angew. Chem. Int. Ed.* **2015**, *54*, 5360-5363.

(34) Jiang, K.; Sun, S.; Zhang, L.; Wang, Y.; Cai, C.; Lin, H., Bright-Yellow-Emissive N-Doped Carbon Dots: Preparation, Cellular Imaging, and Bifunctional Sensing. *ACS Appl. Mater. Interfaces* **2015**, *7*, 23231-23238.

(35) Xu, Y.; Wu, M.; Liu, Y.; Feng, X.-Z.; Yin, X.-B.; He, X.-W.; Zhang, Y.-K., Nitrogen-Doped Carbon Dots: A Facile and General Preparation Method, Photoluminescence Investigation, and Imaging Applications. *Chemistry - A European Journal* **2013**, *19*, 2276-2283.

(36) Qu, K.; Wang, J.; Ren, J.; Qu, X., Carbon Dots Prepared by Hydrothermal Treatment of Dopamine as an Effective Fluorescent Sensing Platform for the Label-Free Detection of Iron(III) Ions and Dopamine. *Chemistry - A European Journal* **2013**, *19*, 7243-7249.

(37) Wang, R.; Wang, X.; Sun, Y., One-step synthesis of self-doped carbon dots with highly photoluminescence as multifunctional biosensors for detection of iron ions and pH. *Sensors and Actuators B: Chemical* **2017**, *241*, 73-79.

(38) Song, Y.; Zhu, C.; Song, J.; Li, H.; Du, D.; Lin, Y., Drug-Derived Bright and Color-Tunable N-Doped Carbon Dots for Cell Imaging and Sensitive Detection of Fe3+ in Living Cells. *ACS Appl. Mater. Interfaces* **2017**, *9*, 7399-7405.

(39) Liu, J.; Lu, S.; Tang, Q.; Zhang, K.; Yu, W.; Sun, H.; Yang, B., One-step hydrothermal synthesis of photoluminescent carbon nanodots with selective antibacterial activity against Porphyromonas gingivalis. *Nanoscale* **2017**, *9*, 7135-7142.

(40) Liu, H.; Li, Z.; Sun, Y.; Geng, X.; Hu, Y.; Meng, H.; Ge, J.; Qu, L., Synthesis of Luminescent Carbon Dots with Ultrahigh Quantum Yield and Inherent Folate Receptor-Positive Cancer Cell Targetability. *Scientific Reports* **2018**, *8*.

(41) Ding, H.; Yu, S.-B.; Wei, J.-S.; Xiong, H.-M., Full-Color Light-Emitting Carbon Dots with a Surface-State-Controlled Luminescence Mechanism. *ACS Nano* **2015**, *10*, 484-491.

(42) Liu, J.; Li, D.; Zhang, K.; Yang, M.; Sun, H.; Yang, B., One-Step Hydrothermal Synthesis of Nitrogen-Doped Conjugated Carbonized Polymer Dots with 31% Efficient Red Emission for In Vivo Imaging. *Small* **2018**, *14*, 1703919.

(43) Ding, H.; Wei, J.-S.; Zhang, P.; Zhou, Z.-Y.; Gao, Q.-Y.; Xiong, H.-M., Solvent-Controlled Synthesis of Highly Luminescent Carbon Dots with a Wide Color Gamut and Narrowed Emission Peak Widths. *Small* **2018**, *14*, 1800612.

(44) Lin, C.; Zhuang, Y.; Li, W.; Zhou, T.-L.; Xie, R.-J., Blue, green, and red full-color ultralong afterglow in nitrogen-doped carbon dots. *Nanoscale* **2019**, *11*, 6584-6590.

(45) Li, G.; Fu, H.; Chen, X.; Gong, P.; Chen, G.; Xia, L.; Wang, H.; You, J.; Wu, Y., Facile and Sensitive Fluorescence Sensing of Alkaline Phosphatase Activity with Photoluminescent Carbon Dots Based on Inner Filter Effect. *Anal. Chem.* **2016**, *88*, 2720-2726.

(46) Lu, S.; Sui, L.; Liu, J.; Zhu, S.; Chen, A.; Jin, M.; Yang, B., Near-Infrared Photoluminescent Polymer-Carbon Nanodots with Two-Photon Fluorescence. *Adv. Mater.* **2017**, *29*, 1603443.

(47) Lu, S.; Xiao, G.; Sui, L.; Feng, T.; Yong, X.; Zhu, S.; Li, B.; Liu, Z.; Zou, B.; Jin, M., Piezochromic carbon dots with two‐photon fluorescence. *Angew. Chem.* **2017**, *129*, 6283-6287.

(48) Gong, P.; Sun, L.; Wang, F.; Liu, X.; Yan, Z.; Wang, M.; Zhang, L.; Tian, Z.; Liu, Z.; You, J., Highly fluorescent N-doped carbon dots with two-photon emission for ultrasensitive detection of tumor marker and visual monitor anticancer drug loading and delivery. *Chem. Eng. J.* **2019**, *356*, 994-1002.

(49) Feng, T.; Zeng, Q.; Lu, S.; Yan, X.; Liu, J.; Tao, S.; Yang, M.; Yang, B., Color-Tunable Carbon Dots Possessing Solid-State Emission for Full-Color Light-Emitting Diodes Applications. *ACS Photonics* **2017**, *5*, 502-510.

(50) Lu, S.; Sui, L.; Wu, M.; Zhu, S.; Yong, X.; Yang, B., Graphitic Nitrogen and High-Crystalline Triggered Strong Photoluminescence and Room-Temperature Ferromagnetism in Carbonized Polymer Dots. *Advanced Science* **2019**, *6*, 1801192.

(51) Yuan, F.; Wang, Z.; Li, X.; Li, Y.; Tan, Z. a.; Fan, L.; Yang, S., Bright Multicolor Bandgap Fluorescent Carbon Quantum Dots for Electroluminescent Light-Emitting Diodes. *Adv. Mater.* **2017**, *29*, 1604436.

(52) Liu, J.; Xu, H.; Xu, Y.; Song, Y.; Lian, J.; Zhao, Y.; Wang, L.; Huang, L.; Ji, H.; Li, H., Graphene quantum dots modified mesoporous graphite carbon nitride with significant enhancement of photocatalytic activity. *Appl. Catal., B* **2017**, *207*, 429-437.

(53) Zhang, R.; Chen, W., Nitrogen-doped carbon quantum dots: Facile synthesis and application as a “turn-off” fluorescent probe for detection of Hg2+ ions. *Biosens. Bioelectron.* **2014**, *55*, 83-90.

(54) Lei, Y.; Yang, C.; Hou, J.; Wang, F.; Min, S.; Ma, X.; Jin, Z.; Xu, J.; Lu, G.; Huang, K.-W., Strongly coupled CdS/graphene quantum dots nanohybrids for highly efficient photocatalytic hydrogen evolution: Unraveling the essential roles of graphene quantum dots. *Appl. Catal., B* **2017**, *216*, 59-69.

(55) Yan, X.; Song, Y.; Zhu, C.; Li, H.; Du, D.; Su, X.; Lin, Y., MnO2 Nanosheet-Carbon Dots Sensing Platform for Sensitive Detection of Organophosphorus Pesticides. *Anal. Chem.* **2018**, *90*, 2618-2624.

(56) Shen, P.; Xia, Y., Synthesis-Modification Integration: One-Step Fabrication of Boronic Acid Functionalized Carbon Dots for Fluorescent Blood Sugar Sensing. *Anal. Chem.* **2014**, *86*, 5323-5329.

(57) Gao, G.; Jiang, Y.-W.; Jia, H.-R.; Yang, J.; Wu, F.-G., On-off-on fluorescent nanosensor for Fe3+ detection and cancer/normal cell differentiation via silicon-doped carbon quantum dots. *Carbon* **2018**, *134*, 232-243.

(58) Wang, F.; Xie, Z.; Zhang, H.; Liu, C.-y.; Zhang, Y.-g., Highly Luminescent Organosilane-Functionalized Carbon Dots. *Adv. Funct. Mater.* **2011**, *21*, 1027-1031.

(59) Han, Y.; Chen, Y.; Feng, J.; Liu, J.; Ma, S.; Chen, X., One-Pot Synthesis of Fluorescent Silicon Nanoparticles for Sensitive and Selective Determination of 2,4,6-Trinitrophenol in Aqueous Solution. *Anal. Chem.* **2017**, *89*, 3001-3008.

(60) Shangguan, J.; Huang, J.; He, D.; He, X.; Wang, K.; Ye, R.; Yang, X.; Qing, T.; Tang, J., Highly Fe3+-Selective Fluorescent Nanoprobe Based on Ultrabright N/P Codoped Carbon Dots and Its Application in Biological Samples. *Anal. Chem.* **2017**, *89*, 7477-7484.

(61) Jiang, K.; Wang, Y.; Cai, C.; Lin, H., Conversion of Carbon Dots from Fluorescence to Ultralong Room-Temperature Phosphorescence by Heating for Security Applications. *Adv. Mater.* **2018**, *30*, 1800783.

(62) Jiang, K.; Wang, Y.; Gao, X.; Cai, C.; Lin, H., Facile, Quick, and Gram-Scale Synthesis of Ultralong-Lifetime Room-Temperature-Phosphorescent Carbon Dots by Microwave Irradiation. *Angew. Chem. Int. Ed.* **2018**, *57*, 6216-6220.

(63) Chandra, S.; Das, P.; Bag, S.; Laha, D.; Pramanik, P., Synthesis, functionalization and bioimaging applications of highly fluorescent carbon nanoparticles. *Nanoscale* **2011**, *3*, 1533.

(64) Gong, X.; Liu, Y.; Yang, Z.; Shuang, S.; Zhang, Z.; Dong, C., An “on-off-on” fluorescent nanoprobe for recognition of chromium(VI) and ascorbic acid based on phosphorus/nitrogen dual-doped carbon quantum dot. *Anal. Chim. Acta* **2017**, *968*, 85-96.

(65) Shi, B.; Su, Y.; Zhang, L.; Huang, M.; Liu, R.; Zhao, S., Nitrogen and Phosphorus Co-Doped Carbon Nanodots as a Novel Fluorescent Probe for Highly Sensitive Detection of Fe3+ in Human Serum and Living Cells. *ACS Appl. Mater. Interfaces* **2016**, *8*, 10717-10725.

(66) Martins, N. C. T.; Ângelo, J.; Girão, A. V.; Trindade, T.; Andrade, L.; Mendes, A., N-doped carbon quantum dots/TiO2 composite with improved photocatalytic activity. *Appl. Catal., B* **2016**, *193*, 67-74.

(67) Li, F.; Li, Y.; Yang, X.; Han, X.; Jiao, Y.; Wei, T.; Yang, D.; Xu, H.; Nie, G., Highly Fluorescent Chiral N-S-Doped Carbon Dots from Cysteine: Affecting Cellular Energy Metabolism. *Angew. Chem. Int. Ed.* **2018**, *57*, 2377-2382.

(68) Li, X.; Lau, S. P.; Tang, L.; Ji, R.; Yang, P., Sulphur doping: a facile approach to tune the electronic structure and optical properties of graphene quantum dots. *Nanoscale* **2014**, *6*, 5323-5328.

(69) Bian, S.; Shen, C.; Qian, Y.; Liu, J.; Xi, F.; Dong, X., Facile synthesis of sulfur-doped graphene quantum dots as fluorescent sensing probes for Ag+ ions detection. *Sensors and Actuators B: Chemical* **2017**, *242*, 231-237.

(70) Qu, D.; Zheng, M.; Du, P.; Zhou, Y.; Zhang, L.; Li, D.; Tan, H.; Zhao, Z.; Xie, Z.; Sun, Z., Highly luminescent S, N co-doped graphene quantum dots with broad visible absorption bands for visible light photocatalysts. *Nanoscale* **2013**, *5*, 12272.

(71) Wang, C.; Xu, Z.; Cheng, H.; Lin, H.; Humphrey, M. G.; Zhang, C., A hydrothermal route to water-stable luminescent carbon dots as nanosensors for pH and temperature. *Carbon* **2015**, *82*, 87-95.

(72) Zhang, M.; Su, R.; Zhong, J.; Fei, L.; Cai, W.; Guan, Q.; Li, W.; Li, N.; Chen, Y.; Cai, L.; Xu, Q., Red/orange dual-emissive carbon dots for pH sensing and cell imaging. *Nano Research* **2019**, *12*, 815-821.

(73) Liu, Y.; Duan, W.; Song, W.; Liu, J.; Ren, C.; Wu, J.; Liu, D.; Chen, H., Red Emission B, N, S-co-Doped Carbon Dots for Colorimetric and Fluorescent Dual Mode Detection of Fe3+ Ions in Complex Biological Fluids and Living Cells. *ACS Appl. Mater. Interfaces* **2017**, *9*, 12663-12672.

(74) Yang, H.; Liu, Y.; Guo, Z.; Lei, B.; Zhuang, J.; Zhang, X.; Liu, Z.; Hu, C., Hydrophobic carbon dots with blue dispersed emission and red aggregation-induced emission. *Nat. Commun.* **2019**, *10*.

(75) Li, F.; Li, T.; Sun, C.; Xia, J.; Jiao, Y.; Xu, H., Selenium‐Doped Carbon Quantum Dots for Free‐Radical Scavenging. *Angew. Chem. Int. Ed.* **2017**, *56*, 9910-9914.

(76) Liu, H.; Sun, Y.; Li, Z.; Yang, J.; Aryee, A. A.; Qu, L.; Du, D.; Lin, Y., Lysosome-targeted carbon dots for ratiometric imaging of formaldehyde in living cells. *Nanoscale* **2019**, *11*, 8458-8463.

(77) Long, P.; Feng, Y.; Cao, C.; Li, Y.; Han, J.; Li, S.; Peng, C.; Li, Z.; Feng, W., Self-Protective Room-Temperature Phosphorescence of Fluorine and Nitrogen Codoped Carbon Dots. *Adv. Funct. Mater.* **2018**, *28*, 1800791.

(78) Qian, Z.; Ma, J.; Shan, X.; Feng, H.; Shao, L.; Chen, J., Highly Luminescent N-Doped Carbon Quantum Dots as an Effective Multifunctional Fluorescence Sensing Platform. *Chemistry - A European Journal* **2014**, *20*, 2254-2263.

(79) Nie, H.; Li, M.; Li, Q.; Liang, S.; Tan, Y.; Sheng, L.; Shi, W.; Zhang, S. X.-A., Carbon Dots with Continuously Tunable Full-Color Emission and Their Application in Ratiometric pH Sensing. *Chem. Mater.* **2014**, *26*, 3104-3112.

(80) Yuan, Y. H.; Liu, Z. X.; Li, R. S.; Zou, H. Y.; Lin, M.; Liu, H.; Huang, C. Z., Synthesis of nitrogen-doping carbon dots with different photoluminescence properties by controlling the surface states. *Nanoscale* **2016**, *8*, 6770-6776.

(81) Gao, W.; Song, H.; Wang, X.; Liu, X.; Pang, X.; Zhou, Y.; Gao, B.; Peng, X., Carbon dots with red emission for sensing of Pt2+, Au3+, and Pd2+ and their bioapplications in vitro and in vivo. *ACS Appl. Mater. Interfaces* **2018**, *10*, 1147-1154.

(82) Shangguan, J.; He, D.; He, X.; Wang, K.; Xu, F.; Liu, J.; Tang, J.; Yang, X.; Huang, J., Label-Free Carbon-Dots-Based Ratiometric Fluorescence pH Nanoprobes for Intracellular pH Sensing. *Anal. Chem.* **2016**, *88*, 7837-7843.

(83) Kooshki, H.; Sobhani-Nasab, A.; Eghbali-Arani, M.; Ahmadi, F.; Ameri, V.; Rahimi-Nasrabadi, M., Eco-friendly synthesis of PbTiO3 nanoparticles and PbTiO3/carbon quantum dots binary nano-hybrids for enhanced photocatalytic performance under visible light. *Sep. Purif. Technol.* **2019**, *211*, 873-881.

(84) Zhou, L.; Lin, Y.; Huang, Z.; Ren, J.; Qu, X., Carbon nanodots as fluorescence probes for rapid, sensitive, and label-free detection of Hg2+and biothiols in complex matrices. *Chem. Commun.* **2012**, *48*, 1147-1149.

(85) Guo, Y.; Wang, Z.; Shao, H.; Jiang, X., Hydrothermal synthesis of highly fluorescent carbon nanoparticles from sodium citrate and their use for the detection of mercury ions. *Carbon* **2013**, *52*, 583-589.

(86) Chen, D.; Zhuang, X.; Zhai, J.; Zheng, Y.; Lu, H.; Chen, L., Preparation of highly sensitive Pt nanoparticles-carbon quantum dots/ionic liquid functionalized graphene oxide nanocomposites and application for H2O2 detection. *Sensors and Actuators B: Chemical* **2018**, *255*, 1500-1506.

(87) Xu, Q.; Pu, P.; Zhao, J.; Dong, C.; Gao, C.; Chen, Y.; Chen, J.; Liu, Y.; Zhou, H., Preparation of highly photoluminescent sulfur-doped carbon dots for Fe (III) detection. *Journal of Materials Chemistry A* **2015**, *3*, 542-546.

(88) Yang, M.; Tang, Q.; Meng, Y.; Liu, J.; Feng, T.; Zhao, X.; Zhu, S.; Yu, W.; Yang, B., Reversible “Off–On” Fluorescence of Zn2+-Passivated Carbon Dots: Mechanism and Potential for the Detection of EDTA and Zn2+. *Langmuir* **2018**, *34*, 7767-7775.

(89) Wu, X.; Song, Y.; Yan, X.; Zhu, C.; Ma, Y.; Du, D.; Lin, Y., Carbon quantum dots as fluorescence resonance energy transfer sensors for organophosphate pesticides determination. *Biosens. Bioelectron.* **2017**, *94*, 292-297.

(90) Jia, Q.; Ge, J.; Liu, W.; Zheng, X.; Chen, S.; Wen, Y.; Zhang, H.; Wang, P., A Magnetofluorescent Carbon Dot Assembly as an Acidic H2O2‐Driven Oxygenerator to Regulate Tumor Hypoxia for Simultaneous Bimodal Imaging and Enhanced Photodynamic Therapy. *Adv. Mater.* **2018**, *30*, 1706090.

(91) Wang, Q.; Li, J.; Tu, X.; Liu, H.; Shu, M.; Si, R.; Ferguson, C. T. J.; Zhang, K. A. I.; Li, R., Single Atomically Anchored Cobalt on Carbon Quantum Dots as Efficient Photocatalysts for Visible Light-Promoted Oxidation Reactions. *Chem. Mater.* **2019**, *32*, 734-743.

(92) Jia, X.; Li, J.; Wang, E., One-pot green synthesis of optically pH-sensitive carbon dots with upconversion luminescence. *Nanoscale* **2012**, *4*, 5572.

(93) Chen, B. B.; Liu, Z. X.; Zou, H. Y.; Huang, C. Z., Highly selective detection of 2,4,6-trinitrophenol by using newly developed terbium-doped blue carbon dots. *The Analyst* **2016**, *141*, 2676-2681.

(94) Bourlinos, A. B.; Bakandritsos, A.; Kouloumpis, A.; Gournis, D.; Krysmann, M.; Giannelis, E. P.; Polakova, K.; Safarova, K.; Hola, K.; Zboril, R., Gd(iii)-doped carbon dots as a dual fluorescent-MRI probe. *J. Mater. Chem.* **2012**, *22*, 23327.

(95) Li, X.; Liu, Y.; Song, X.; Wang, H.; Gu, H.; Zeng, H., Intercrossed Carbon Nanorings with Pure Surface States as Low-Cost and Environment-Friendly Phosphors for White-Light-Emitting Diodes. *Angew. Chem. Int. Ed.* **2015**, *54*, 1759-1764.

(96) Jaiswal, A.; Ghosh, S. S.; Chattopadhyay, A., One step synthesis of C-dots by microwave mediated caramelization of poly(ethylene glycol). *Chem. Commun.* **2012**, *48*, 407-409.

(97) Yao, W.; Wang, X.; Liang, Y.; Yu, S.; Gu, P.; Sun, Y.; Xu, C.; Chen, J.; Hayat, T.; Alsaedi, A.; Wang, X., Synthesis of novel flower-like layered double oxides/carbon dots nanocomposites for U(VI) and 241Am(III) efficient removal: Batch and EXAFS studies. *Chem. Eng. J.* **2018**, *332*, 775-786.

(98) Briscoe, J.; Marinovic, A.; Sevilla, M.; Dunn, S.; Titirici, M., Biomass-Derived Carbon Quantum Dot Sensitizers for Solid-State Nanostructured Solar Cells. *Angew. Chem. Int. Ed.* **2015**, *54*, 4463-4468.

(99) Tao, S.; Lu, S.; Geng, Y.; Zhu, S.; Redfern, S. A. T.; Song, Y.; Feng, T.; Xu, W.; Yang, B., Design of Metal-Free Polymer Carbon Dots: A New Class of Room-Temperature Phosphorescent Materials. *Angew. Chem. Int. Ed.* **2018**, *57*, 2393-2398.

(100) Chen, Y.; Zheng, M.; Xiao, Y.; Dong, H.; Zhang, H.; Zhuang, J.; Hu, H.; Lei, B.; Liu, Y., A Self-Quenching-Resistant Carbon-Dot Powder with Tunable Solid-State Fluorescence and Construction of Dual-Fluorescence Morphologies for White Light-Emission. *Adv. Mater.* **2016**, *28*, 312-318.

(101) Ge, J.; Jia, Q.; Liu, W.; Guo, L.; Liu, Q.; Lan, M.; Zhang, H.; Meng, X.; Wang, P., Red-Emissive Carbon Dots for Fluorescent, Photoacoustic, and Thermal Theranostics in Living Mice. *Adv. Mater.* **2015**, *27*, 4169-4177.

(102) Ge, J.; Lan, M.; Zhou, B.; Liu, W.; Guo, L.; Wang, H.; Jia, Q.; Niu, G.; Huang, X.; Zhou, H., A graphene quantum dot photodynamic therapy agent with high singlet oxygen generation. *Nat. Commun.* **2014**, *5*, 1-8.

(103) Dong, Y.; Wang, R.; Li, G.; Chen, C.; Chi, Y.; Chen, G., Polyamine-Functionalized Carbon Quantum Dots as Fluorescent Probes for Selective and Sensitive Detection of Copper Ions. *Anal. Chem.* **2012**, *84*, 6220-6224.

(104) Liu, M.; Ji, J.; Zhang, X.; Zhang, X.; Yang, B.; Deng, F.; Li, Z.; Wang, K.; Yang, Y.; Wei, Y., Self-polymerization of dopamine and polyethyleneimine: novel fluorescent organic nanoprobes for biological imaging applications. *Journal of Materials Chemistry B* **2015**, *3*, 3476-3482.

(105) Zhang, H.; Chen, Y.; Liang, M.; Xu, L.; Qi, S.; Chen, H.; Chen, X., Solid-Phase Synthesis of Highly Fluorescent Nitrogen-Doped Carbon Dots for Sensitive and Selective Probing Ferric Ions in Living Cells. *Anal. Chem.* **2014**, *86*, 9846-9852.

(106) Liu, C.; Zhang, P.; Zhai, X.; Tian, F.; Li, W.; Yang, J.; Liu, Y.; Wang, H.; Wang, W.; Liu, W., Nano-carrier for gene delivery and bioimaging based on carbon dots with PEI-passivation enhanced fluorescence. *Biomaterials* **2012**, *33*, 3604-3613.

(107) Dou, Q.; Fang, X.; Jiang, S.; Chee, P. L.; Lee, T.-C.; Loh, X. J., Multi-functional fluorescent carbon dots with antibacterial and gene delivery properties. *RSC Advances* **2015**, *5*, 46817-46822.

(108) Guo, X.; Wang, C.-F.; Yu, Z.-Y.; Chen, L.; Chen, S., Facile access to versatile fluorescent carbon dots toward light-emitting diodes. *Chem. Commun.* **2012**, *48*, 2692.

(109) Lan, M.; Zhao, S.; Zhang, Z.; Yan, L.; Guo, L.; Niu, G.; Zhang, J.; Zhao, J.; Zhang, H.; Wang, P.; Zhu, G.; Lee, C.-S.; Zhang, W., Two-photon-excited near-infrared emissive carbon dots as multifunctional agents for fluorescence imaging and photothermal therapy. *Nano Research* **2017**, *10*, 3113-3123.

(110) Gong, X.; Lu, W.; Paau, M. C.; Hu, Q.; Wu, X.; Shuang, S.; Dong, C.; Choi, M. M. F., Facile synthesis of nitrogen-doped carbon dots for Fe3+ sensing and cellular imaging. *Anal. Chim. Acta* **2015**, *861*, 74-84.

(111) Hu, S.; Trinchi, A.; Atkin, P.; Cole, I., Tunable Photoluminescence Across the Entire Visible Spectrum from Carbon Dots Excited by White Light. *Angew. Chem. Int. Ed.* **2015**, *54*, 2970-2974.

(112) Lin, Z.; Xue, W.; Chen, H.; Lin, J.-M., Peroxynitrous-acid-induced chemiluminescence of fluorescent carbon dots for nitrite sensing. *Anal. Chem.* **2011**, *83*, 8245-8251.

(113) Hu, X.; Shi, J.; Shi, Y.; Zou, X.; Arslan, M.; Zhang, W.; Huang, X.; Li, Z.; Xu, Y., Use of a smartphone for visual detection of melamine in milk based on Au@Carbon quantum dots nanocomposites. *Food Chem.* **2019**, *272*, 58-65.

(114) Liu, S.; Tian, J.; Wang, L.; Zhang, Y.; Qin, X.; Luo, Y.; Asiri, A. M.; Al-Youbi, A. O.; Sun, X., Hydrothermal Treatment of Grass: A Low-Cost, Green Route to Nitrogen-Doped, Carbon-Rich, Photoluminescent Polymer Nanodots as an Effective Fluorescent Sensing Platform for Label-Free Detection of Cu(II) Ions. *Adv. Mater.* **2012**, *24*, 2037-2041.

(115) Lu, W.; Qin, X.; Liu, S.; Chang, G.; Zhang, Y.; Luo, Y.; Asiri, A. M.; Al-Youbi, A. O.; Sun, X., Economical, green synthesis of fluorescent carbon nanoparticles and their use as probes for sensitive and selective detection of mercury (II) ions. *Anal. Chem.* **2012**, *84*, 5351-5357.

(116) Zhu, C.; Zhai, J.; Dong, S., Bifunctional fluorescent carbon nanodots: green synthesis via soy milk and application as metal-free electrocatalysts for oxygen reduction. *Chem. Commun.* **2012**, *48*, 9367.

(117) Sahu, S.; Behera, B.; Maiti, T. K.; Mohapatra, S., Simple one-step synthesis of highly luminescent carbon dots from orange juice: application as excellent bio-imaging agents. *Chem. Commun.* **2012**, *48*, 8835.

(118) Hsu, P.-C.; Shih, Z.-Y.; Lee, C.-H.; Chang, H.-T., Synthesis and analytical applications of photoluminescent carbon nanodots. *Green Chemistry* **2012**, *14*, 917.

(119) De, B.; Karak, N., A green and facile approach for the synthesis of water soluble fluorescent carbon dots from banana juice. *RSC Advances* **2013**, *3*, 8286.

(120) Li, W.; Zhang, Z.; Kong, B.; Feng, S.; Wang, J.; Wang, L.; Yang, J.; Zhang, F.; Wu, P.; Zhao, D., Simple and Green Synthesis of Nitrogen-Doped Photoluminescent Carbonaceous Nanospheres for Bioimaging. *Angew. Chem. Int. Ed.* **2013**, *52*, 8151-8155.

(121) Liang, Q.; Ma, W.; Shi, Y.; Li, Z.; Yang, X., Easy synthesis of highly fluorescent carbon quantum dots from gelatin and their luminescent properties and applications. *Carbon* **2013**, *60*, 421-428.

(122) Yang, X.; Zhuo, Y.; Zhu, S.; Luo, Y.; Feng, Y.; Dou, Y., Novel and green synthesis of high-fluorescent carbon dots originated from honey for sensing and imaging. *Biosens. Bioelectron.* **2014**, *60*, 292-298.

(123) Wang, Q.; Huang, X.; Long, Y.; Wang, X.; Zhang, H.; Zhu, R.; Liang, L.; Teng, P.; Zheng, H., Hollow luminescent carbon dots for drug delivery. *Carbon* **2013**, *59*, 192-199.

(124) Wang, L.; Zhou, H. S., Green Synthesis of Luminescent Nitrogen-Doped Carbon Dots from Milk and Its Imaging Application. *Anal. Chem.* **2014**, *86*, 8902-8905.

(125) Mehta, V. N.; Jha, S.; Basu, H.; Singhal, R. K.; Kailasa, S. K., One-step hydrothermal approach to fabricate carbon dots from apple juice for imaging of mycobacterium and fungal cells. *Sensors and Actuators B: Chemical* **2015**, *213*, 434-443.

(126) Xu, H.; Yang, X.; Li, G.; Zhao, C.; Liao, X., Green Synthesis of Fluorescent Carbon Dots for Selective Detection of Tartrazine in Food Samples. *J. Agric. Food. Chem.* **2015**, *63*, 6707-6714.

(127) Sachdev, A.; Gopinath, P., Green synthesis of multifunctional carbon dots from coriander leaves and their potential application as antioxidants, sensors and bioimaging agents. *The Analyst* **2015**, *140*, 4260-4269.

(128) Wang, N.; Wang, Y.; Guo, T.; Yang, T.; Chen, M.; Wang, J., Green preparation of carbon dots with papaya as carbon source for effective fluorescent sensing of Iron (III) and Escherichia coli. *Biosens. Bioelectron.* **2016**, *85*, 68-75.

(129) Edison, T. N. J. I.; Atchudan, R.; Shim, J.-J.; Kalimuthu, S.; Ahn, B.-C.; Lee, Y. R., Turn-off fluorescence sensor for the detection of ferric ion in water using green synthesized N-doped carbon dots and its bio-imaging. *Journal of Photochemistry and Photobiology B: Biology* **2016**, *158*, 235-242.

(130) Atchudan, R.; Edison, T. N. J. I.; Aseer, K. R.; Perumal, S.; Karthik, N.; Lee, Y. R., Highly fluorescent nitrogen-doped carbon dots derived from Phyllanthus acidus utilized as a fluorescent probe for label-free selective detection of Fe3+ ions, live cell imaging and fluorescent ink. *Biosens. Bioelectron.* **2018**, *99*, 303-311.

(131) Li, W.; Liu, Y.; Wu, M.; Feng, X.; Redfern, S. A. T.; Shang, Y.; Yong, X.; Feng, T.; Wu, K.; Liu, Z.; Li, B.; Chen, Z.; Tse, J. S.; Lu, S.; Yang, B., Carbon-Quantum-Dots-Loaded Ruthenium Nanoparticles as an Efficient Electrocatalyst for Hydrogen Production in Alkaline Media. *Adv. Mater.* **2018**, *30*, 1800676.

(132) Zhao, S.; Lan, M.; Zhu, X.; Xue, H.; Ng, T.-W.; Meng, X.; Lee, C.-S.; Wang, P.; Zhang, W., Green Synthesis of Bifunctional Fluorescent Carbon Dots from Garlic for Cellular Imaging and Free Radical Scavenging. *ACS Appl. Mater. Interfaces* **2015**, *7*, 17054-17060.

(133) Long, R.; Guo, Y.; Xie, L.; Shi, S.; Xu, J.; Tong, C.; Lin, Q.; Li, T., White pepper-derived ratiometric carbon dots for highly selective detection and imaging of coenzyme A. *Food Chem.* **2020**, *315*, 126171.

(134) Li, W.; Wei, Z.; Wang, B.; Liu, Y.; Song, H.; Tang, Z.; Yang, B.; Lu, S., Carbon quantum dots enhanced the activity for the hydrogen evolution reaction in ruthenium-based electrocatalysts. *Mater. Chem. Front.* **2020**, *4*, 277-284.

(135) Qi, H.; Teng, M.; Liu, M.; Liu, S.; Li, J.; Yu, H.; Teng, C.; Huang, Z.; Liu, H.; Shao, Q.; Umar, A.; Ding, T.; Gao, Q.; Guo, Z., Biomass-derived nitrogen-doped carbon quantum dots: highly selective fluorescent probe for detecting Fe3+ ions and tetracyclines. *J. Colloid Interface Sci.* **2019**, *539*, 332-341.
